# Genome-Resolved Metagenomics Reveals Thermophilic Microbial Diversity and Putative Hydrolase-Encoding Genes in the El Tatio Geothermal Field

**DOI:** 10.3390/ijms27156905

**Published:** 2026-08-01

**Authors:** Bernardita Valenzuela, Ignacio Navarrete-Diaz, Mayra Cayo, Francisco Solís-Cornejo, Pedro Zamorano

**Affiliations:** 1 Departamento de Educación, Facultad de Educación, Universidad de Antofagasta, Antofagasta 1240000, Chile; 2 Laboratorio de Microorganismos Extremófilos, Instituto Antofagasta, Universidad de Antofagasta, Antofagasta 1240000, Chile; ignacio.navarrete.diaz@ua.cl (I.N.-D.); mayra.cayo@uamail.cl (M.C.); francisco.solis@uamail.cl (F.S.-C.); 3 Programa de Doctorado en Ciencias Biológicas de Ambientes Extremos, Facultad de Ciencias de la Salud y Facultad de Ciencias del Mar y Recursos Biológicos, Universidad de Antofagasta, Antofagasta 1240000, Chile; 4 Programa de Doctorado en Ciencias Aplicadas Mención Sistemas Acuáticos, Facultad de Ciencias del Mar y Recursos Biológicos, Universidad de Antofagasta, Antofagasta 1240000, Chile; 5 Departamento Biomédico, Facultad de Ciencias de la Salud, Universidad de Antofagasta, Antofagasta 1240000, Chile

**Keywords:** shotgun metagenomics, metagenome-assembled genomes (MAGs), thermophilic microorganisms, thermostable hydrolases, enzyme bioprospecting

## Abstract

Geothermal ecosystems constitute important reservoirs of thermophilic microorganisms and their associated metabolic functions; however, the genome-resolved diversity and enzymatic potential of high-altitude geothermal systems remain poorly characterized. Here, we applied shotgun metagenomics and genome-resolved approaches to investigate thermophilic microbial communities inhabiting geothermal sediments from the El Tatio geothermal field, a polyextreme hydrothermal system located at ~4300 m above sea level in the Andean Altiplano of northern Chile. Genome reconstruction yielded 657 metagenome-assembled genomes (MAGs), including 190 near-complete and 273 high-quality genomes, providing a comprehensive genome-resolved view of microbial diversity in this environment. Taxonomic analyses revealed diverse archaeal and bacterial communities dominated by members of *Thermoproteota*, *Methanobacteriota*, *Deinococcota*, and *Actinomycetota*. Functional screening identified 612 high-confidence putative hydrolase-encoding genes distributed across multiple thermophilic lineages, including genes associated with esterases, lipases, proteases, and glycoside hydrolases. Notably, several candidates were recovered from archaeal MAGs affiliated with *Thermoproteus*, *Sulfolobales*, *Pyrobaculum*, and *Acidilobaceae*, expanding the genomic repertoire of putative hydrolytic functions in thermophilic archaea. Sequence-based thermostability prediction identified proteins with estimated melting temperatures exceeding 80 °C, with the highest predicted value reaching 87.6 °C. Collectively, these results expand current knowledge of microbial diversity and functional potential in high-altitude geothermal ecosystems and identify El Tatio as a rich source of putative hydrolase-encoding genes for future biochemical and biotechnological exploration.

## 1. Introduction

Geothermal ecosystems harbor thermophilic microorganisms adapted to extreme physicochemical conditions and constitute important reservoirs of genetic and metabolic diversity. Elevated temperatures, steep redox gradients, variable pH, fluctuating water availability, and geochemical heterogeneity impose strong selective pressures that shape highly specialized microbial communities and drive the evolution of proteins with enhanced structural stability and catalytic performance. Among these, thermostable enzymes have attracted considerable attention because of their ability to maintain activity under harsh industrial conditions, making them valuable for applications in biorefineries, polymer processing, food production, detergents, and biopharmaceutical manufacturing [1,2]. Consequently, geothermal microbiomes have become major targets for the discovery of novel enzymatic functions and thermostable biocatalytic systems [3,4].

Recent advances in shotgun metagenomics have transformed the exploration of microbial life in extreme environments by enabling direct access to the genetic repertoire of entire microbial communities without the need for cultivation. Beyond taxonomic profiling, genome-resolved metagenomics allows the reconstruction of metagenome-assembled genomes (MAGs), providing the opportunity to link metabolic potential and functional traits to specific microbial lineages [5,6]. This approach has substantially expanded our understanding of microbial diversity in geothermal ecosystems, revealing previously uncultivated archaeal and bacterial lineages as well as novel metabolic capabilities associated with adaptation to extreme environmental conditions [6,7]. High-altitude geothermal systems represent particularly valuable environments for studying microbial adaptation because they combine thermal stress with additional environmental pressures, including reduced atmospheric pressure, low oxygen availability, intense ultraviolet radiation, and pronounced diel temperature fluctuations. These polyextreme conditions generate unique selective regimes that may promote distinctive evolutionary and metabolic adaptations compared with geothermal systems located at lower elevations [8,9]. As a result, high-altitude hydrothermal environments have emerged as important natural laboratories for investigating microbial evolution under multiple simultaneous stressors and for exploring the genetic basis of adaptation to extreme conditions.

The El Tatio geothermal field, located in the Andean Altiplano of northern Chile at approximately 4300 m above sea level, is one of the highest geothermal systems on Earth. This hydrothermal field comprises numerous geysers, hot springs, microbial mats, and geothermal sediments that support diverse thermophilic microbial communities and have been recognized as environments of geobiological and astrobiological relevance. Previous studies have documented microbial signatures preserved in hydrothermal deposits and demonstrated the influence of local physicochemical gradients on microbial community structure [9,10]. However, despite increasing interest in El Tatio as a model extreme environment, genome-resolved investigations exploring the diversity and functional potential of sediment-associated microbial communities remain limited.

Among the functional traits of biotechnological interest in thermophilic microorganisms, hydrolytic enzymes play central roles in the degradation of complex organic substrates and are widely exploited in industrial processes. Thermostable hydrolases, including esterases, lipases, proteases, and glycoside hydrolases, are particularly attractive because of their stability and catalytic performance at elevated temperatures [1].

Genome-resolved metagenomics offers an effective strategy for identifying genes encoding these enzymes directly from environmental genomes and for linking their distribution to specific archaeal and bacterial lineages [4,5]. In this study, we applied shotgun metagenomics and genome-resolved analyses to characterize thermophilic microbial communities inhabiting geothermal sediments from the El Tatio geothermal field. Specifically, we reconstructed metagenome-assembled genomes to investigate microbial diversity and examine the distribution of putative hydrolase-encoding genes across archaeal and bacterial thermophilic lineages, providing a genome-resolved framework for understanding the enzymatic potential of this high-altitude geothermal ecosystem.

## 2. Results

### 2.1. Study Site Characterization and Sample Overview

**Figure 1:**
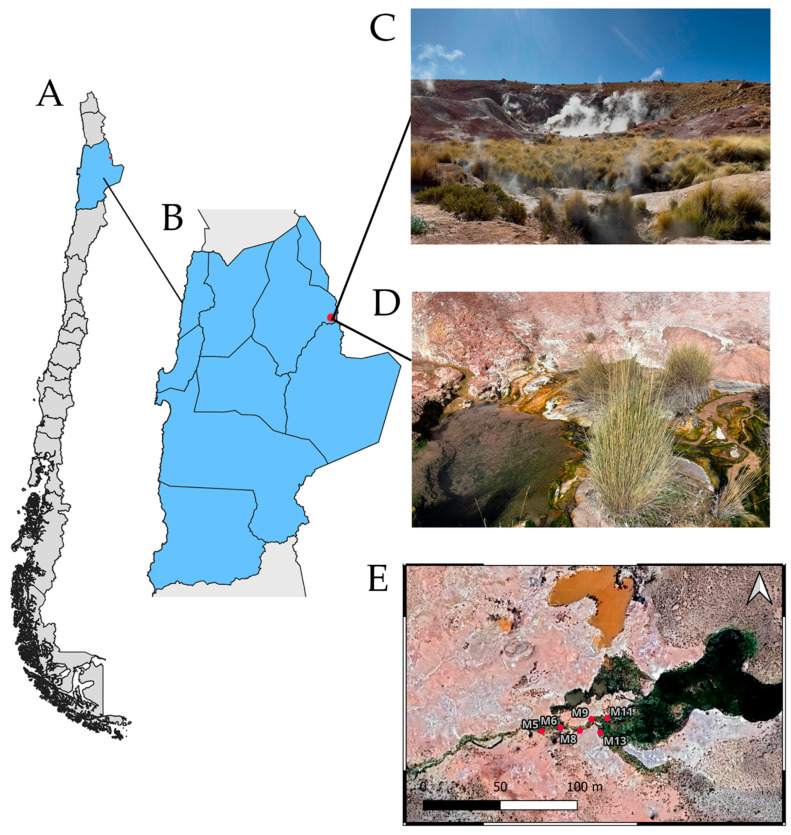
Geographic context of the El Tatio geothermal field. (**A**) Location map showing Chile and the Antofagasta Region (highlighted in blue). (**B**) Map of the Antofagasta Region indicating the location of the El Tatio geothermal field (red dot). (**C**) General view of the sampling area within the geothermal field. (**D**) Sampling site showing orange-colored microbial mats associated with geothermal sediments. (**E**) Detailed map of the sampling area, showing the precise locations of the sediment samples collected.

Geothermal sediment and microbial mat samples were collected from six thermally active locations within the El Tatio geothermal field in the Andean Altiplano of northern Chile (Figure 1). In situ temperatures ranged from 70 to 82 °C, while pH values ranged from 3.80 to 6.79 where measurements were available (Table 1). The sampled environments included exposed geothermal sediments and microbial mats associated with hydrothermal outflows, representing distinct geothermal microenvironments for the investigation of thermophilic microbial communities.

### 2.2. DNA Yield and Shotgun Metagenomic Sequencing Output

High-quality environmental DNA was successfully recovered from all samples, with concentrations ranging from 1.29 to 9.44 ng/µL (Table 2). All DNA extracts met quality requirements for shotgun metagenomic sequencing.

Sequencing generated between 12.0 and 12.9 Gbp per sample, corresponding to 79.6–85.4 million paired-end reads (Table 3). GC content ranged from 53.1% to 60.5% across samples. These datasets were subsequently used for taxonomic profiling, metagenomic assembly, genome reconstruction, and functional analyses.

### 2.3. Taxonomic Composition of Thermophilic Microbial Communities

**Figure 2:**
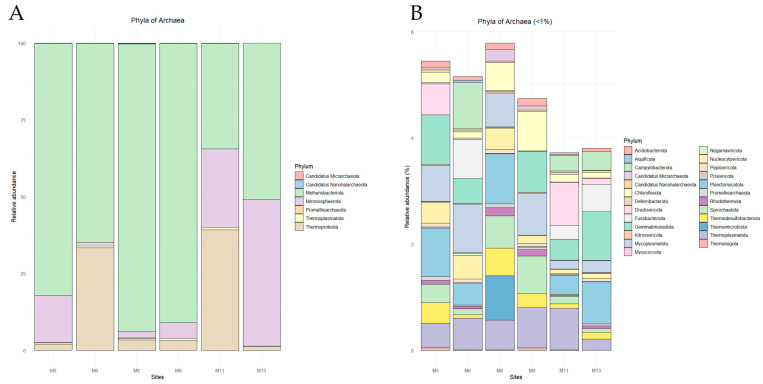
Relative abundance of archaeal phyla across the El Tatio geothermal samples. (**A**) Relative abundance of archaeal phyla. (**B**) Relative abundance of archaeal phyla under 1%. Bars represent the relative abundance of each phylum within individual samples.

**Figure 3:**
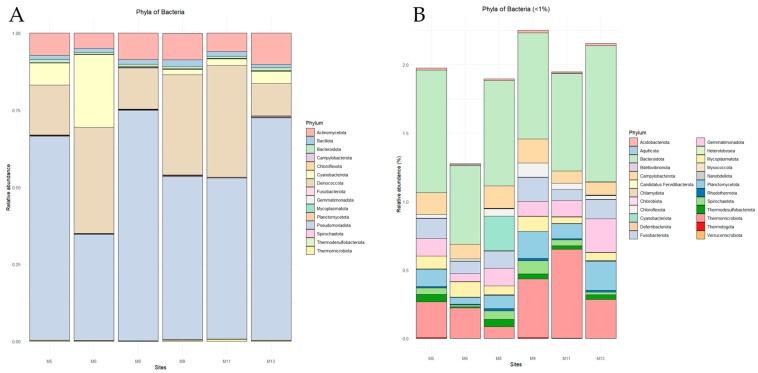
Bacteria phylum-level taxonomic composition. (**A**) Relative abundance of bacterial phyla. (**B**) Relative abundance of bacterial phyla with <1% abundance. Bars represent the relative abundance of each phylum within individual samples.

Taxonomic profiling of quality-filtered metagenomic reads revealed diverse archaeal and bacterial communities inhabiting the geothermal sediments of the El Tatio geothermal field (Figure 2 and Figure 3). Classification using Kraken2 showed variable taxonomic assignment across samples, with classified reads ranging from 21.70% to 63.79%, whereas unclassified reads ranged from 36.21% to 78.30% (Table 4). Except for sample M6, most metagenomes were dominated by unclassified reads, indicating that a substantial fraction of the recovered sequences could not be assigned to reference taxa under the classification criteria used [5,6,11].

At the phylum level, the dominant lineages included *Thermoproteota*, *Methanobacteriota*, *Deinococcota*, and *Actinomycetota* [9,12].

#### 2.3.1. Archaeal Community Structure

Archaeal reads were assigned to seven phyla across all samples (Figure 2). *Thermoproteota*, *Methanobacteriota*, and *Nitrososphaerota* represented the dominant archaeal lineages throughout the dataset.

Several low-abundance archaeal groups (<1% relative abundance) were also detected (Figure 2B). *Promethearchaeota* were identified in all samples (M5, M6, M8, M9, M11, and M13), whereas *Thermoplasmatota* were detected in samples M5, M6, M9, and M13. *Nanoarchaeota* were restricted to samples M8 and M11, while *Woesearchaeota* and *Aenigmarchaeota* were detected exclusively in samples M6 and M9, respectively. Together, these results reveal the coexistence of dominant thermophilic archaeal taxa and a diverse low-abundance archaeal component across the sampled geothermal sediments [13,14].

#### 2.3.2. Bacterial Community Structure

Bacterial communities exhibited substantial phylogenetic diversity across the geothermal sediment samples (Figure 3). The dominant bacterial phyla were primarily Pseudomonadota, followed by *Cyanobacteriota*, and also included *Deinococcota* and *Actinomycetota* [12,15].

Several low-abundance bacterial phyla (<1% relative abundance) were also identified (Figure 3B). Among these, *Aquificota*, *Thermotogota*, *Thermodesulfobacteriota*, and *Thermomicrobiota* were detected in multiple samples, together with *Deferribacterota*, *Rhodothermia*, *Fusobacteriota*, and *Verrucomicrobiota*.

Overall, the bacterial communities comprised both dominant thermophilic lineages and a diverse low-abundance fraction [12,16].

### 2.4. Metagenomic Assembly and Gene Prediction

Metagenomic assembly generated a large number of contigs across all samples, including 497,016 contigs longer than 1 kb (Table 5). The number of assembled contigs per sample ranged from 43,207 to 110,121, while N50 values varied between 4424 and 15,404 bp, indicating successful recovery of medium-length contigs suitable for downstream genome-resolved analyses.

Gene prediction identified a total of 4,640,864 open reading frames (ORFs) across the six metagenomes, with individual samples containing between 408,018 and 970,453 predicted genes (Table 5). Preliminary similarity-based screening against the thermophile-derived hydrolase reference database identified 878,934 hydrolase-like matches prior to filtering.

Together, the assembled contigs and predicted gene catalogs provided the basis for subsequent genome binning, reconstruction of metagenome-assembled genomes (MAGs), and identification of putative hydrolase-encoding genes.

Hydrolase-like matches correspond to raw sequence similarity hits identified against the thermophile-derived hydrolase reference database prior to filtering based on sequence identity and protein alignment length thresholds.

### 2.5. Identification of Putative Hydrolase-Encoding Genes

Functional screening of the metagenomic assemblies revealed a large repertoire of genes encoding putative hydrolases across all geothermal sediment samples. Initial similarity searches against the curated thermophile-derived hydrolase reference database identified 878,934 hydrolase-like matches across the six metagenomes (Table 5). To obtain a conservative set of candidates, DIAMOND matches were filtered using thresholds of ≥60% amino-acid identity and ≥250 amino acids alignment length, reducing the dataset to 612 high-confidence putative hydrolase-encoding genes.

The filtered dataset comprised multiple enzyme classes commonly associated with thermophilic microbial metabolism, including esterases, lipases, proteases, and polysaccharide-degrading enzymes. Esterases and lipases represented the most abundant categories across the metagenomes, followed by proteases, whereas polysaccharide-degrading enzymes were detected at lower frequencies (Table 6). The distribution of hydrolase classes varied among samples, indicating differences in the functional potential encoded within individual geothermal communities.

Predicted thermostability analysis revealed marked differences among enzyme classes. Esterases exhibited the highest mean predicted melting temperature (81.1 °C), closely followed by lipases (79.9 °C) and other hydrolases (79.8 °C), whereas proteases displayed intermediate values (74.9 °C). In contrast, pectinases showed substantially lower predicted thermostability, with a mean predicted melting temperature of 53.8 °C (Table 6). Several individual candidates exhibited predicted melting temperatures above 85 °C, with the highest predicted value reaching 87.6 °C.

Collectively, these results indicate that the El Tatio geothermal sediments harbor a diverse repertoire of putative hydrolase-encoding genes spanning multiple enzyme classes and thermal stability profiles.

Values represent the mean ± standard deviation calculated across the six metagenomic samples (M5, M6, M8, M9, M11, and M13).

### 2.6. Reconstruction and Quality Assessment of Metagenome-Assembled Genomes (MAGs)

Genome-resolved metagenomic reconstruction yielded a total of 657 metagenome-assembled genomes (MAGs) across the six geothermal sediment samples (Table 7). The number of reconstructed genomes per sample ranged from 57 in sample M6 to 131 in sample M11, reflecting variation in microbial community complexity and assembly outcomes among sampling sites.

Genome quality was assessed using CheckM according to MIMAG criteria based on estimates of completeness and contamination (Supplementary Figure S2). Among the reconstructed genomes, 430 met the criteria for medium-quality MAGs (≥50% completeness and <10% contamination), including 273 high-quality MAGs (≥90% completeness and <5% contamination) and 190 near-complete genomes (≥95% completeness and <5% contamination). Notably, ten MAGs achieved 100% estimated completeness while maintaining contamination levels below 5%.

The distribution of marker genes used for genome quality assessment is shown in Supplementary Figure S2. Across all samples, the majority of reconstructed genomes displayed high completeness and low contamination values.

Taxonomic classification of the recovered MAGs revealed a diverse assemblage of thermophilic archaeal and bacterial lineages, including representatives of *Thermoproteota*, *Methanobacteriota*, *Deinococcota*, and *Actinomycetota*. The near-complete MAGs, including completeness, contamination and closest GTDB-Tk classifications, are provided in Supplementary Table S1.

### 2.7. Distribution of Hydrolase-Encoding Genes Across MAGs

The 612 high-confidence putative hydrolase-encoding genes identified in the metagenomic datasets were unevenly distributed across the reconstructed metagenome-assembled genomes (MAGs). While many genomes contained only a limited number of hydrolase-associated sequences, a subset of MAGs concentrated multiple candidates.

Hydrolase-encoding genes were detected across both bacterial and archaeal lineages. Bacterial MAGs affiliated with thermophilic taxa accounted for the majority of hydrolase-rich genomes, including representatives of *Meiothermus*, *Thermus*, *Calidithermus*, and *Caldilinea*. Several archaeal MAGs also encoded putative hydrolases, including genomes assigned to *Thermoproteus*, *Sulfolobales*, *Pyrobaculum*, and *Acidilobaceae*, extending the distribution of hydrolytic functions across diverse thermophilic archaeal lineages.

Representative hydrolase-rich MAGs selected according to genome quality, hydrolase gene content, predicted thermostability, and taxonomic diversity are summarized in Table 8 and Supplementary Table S2. Although hydrolytic potential was concentrated in a subset of genomes, the presence of hydrolase-associated genes was across phylogenetically diverse bacterial and archaeal taxa.

These results demonstrate that genome-resolved reconstruction enables the linkage of putative hydrolase-encoding genes to specific microbial lineages.

### 2.8. Identification of Putative Thermostable Enzyme Candidates

Analysis of the predicted physicochemical properties of the selected hydrolase candidates revealed a broad range of thermal stability profiles across the reconstructed genomes. Predicted melting temperatures (Tm) ranged from approximately 61 °C to 87.6 °C, indicating substantial variation in thermostability among the identified proteins (Table 8).

Several candidates exhibited particularly high predicted thermostability, with estimated melting temperatures exceeding 80 °C. The highest predicted value (87.6 °C) was identified in a carbon–nitrogen hydrolase encoded by a MAG affiliated with *Thermus antranikianii* (M6 43ccT); however, this same temperature has been identified in other microorganisms such as *Meiothermus_B silvanus* (M11 29mtB). Additional highly thermostable candidates included Zn-dependent hydrolases (85.1 °C), alpha/beta-fold hydrolases (85.0 °C), serine proteases (84.9 °C), and glycoside hydrolases (84.5 °C), all recovered from thermophilic bacterial lineages.

MAGs 60. amino-acid identity and alignment lengths ≥ 250 amino acids. Archaeal MAGs with fewer hydrolase-associated sequences were retained when they represented underexplored taxonomic groups, thereby capturing a broader spectrum of potential enzymatic diversity within the El Tatio geothermal microbiome. Hydrolase candidates were distributed across multiple bacterial and archaeal taxa. Bacterial MAGs affiliated with *Thermus*, *Meiothermus*, *Calidithermus*, and *Caldilinea* contained the majority of highly thermostable candidates, whereas archaeal MAGs assigned to *Thermoproteus*, *Sulfolobales*, *Pyrobaculum*, and Acidilobaceae encoded a smaller but phylogenetically diverse repertoire of putative hydrolases. Notably, archaeal candidates included ATP-dependent proteases and S-adenosyl-L-homocysteine hydrolases with predicted melting temperatures approaching 80 °C.

Representative candidates selected on the basis of predicted thermostability, functional annotation, and genomic context are summarized in Table 8. Together, these results highlight the diversity of putative thermostable hydrolases encoded within the El Tatio geothermal microbiome and provide a prioritized set of candidates for future biochemical and structural characterization.

### 2.9. Functional Assignment of Selected Putative Thermostable Hydrolases

To refine the functional annotation of selected hydrolase candidates with high predicted melting temperatures, representative sequences were further analyzed using BLASTp against the NCBI clustered non-redundant protein database and KOfamKO annotation against KEGG Ortholog Profiles (Table S3). These analyses were performed to complement the initial similarity-based screening against the custom thermophile-derived hydrolase database and to provide additional support for functional assignments.

BLASTp searches revealed similarity to diverse hydrolytic and hydrolase-related protein families, including glycoside hydrolases, alpha/beta-fold hydrolases, carbon-nitrogen hydrolases, metallohydrolases, peptidases, and ATP-dependent proteases. Several candidates recovered from thermophilic bacterial lineages, particularly members of *Meiothermus*, *Thermus*, *Calidithermus*, and *Caldilinea*, exhibited high sequence identities frequently exceeding 90%, supporting their annotation as putative hydrolases.

KOfamKO analyses provided additional support for several candidates through significant matches to KEGG Orthologs associated with alpha-glucosidases, glucoamylases, cyclomaltodextrinase/maltogenic alpha-amylase/neopullulanase proteins, N-carbamoylputrescine amidases, metallohydrolases, and ATP-dependent proteases. However, not all annotations were fully concordant between BLASTp and KOfamKO, and several proteins remained assigned to broad functional categories or uncharacterized protein families.

Taken together, these analyses support the putative hydrolytic functions of the selected candidates while highlighting the need for experimental validation to confirm enzyme activity and substrate specificity.

## 3. Discussion

The present study provides a comprehensive genome-resolved characterization of thermophilic microbial communities inhabiting geothermal sediments of the Andean Altiplano and highlights their potential as reservoirs of thermostable enzymes with biotechnological relevance. By integrating shotgun metagenomics with metagenome-assembled genome (MAG) reconstruction, this work advances current understanding of how extreme environmental pressures shape microbial diversity, metabolic potential, and functional specialization in high-altitude geothermal systems.

### 3.1. Thermophilic Community Structure in High-Altitude Geothermal Sediments

Microbial communities inhabiting geothermal sediments of the Andean Altiplano exhibit a pronounced thermophilic signature shaped by high temperature, geochemical gradients, and persistent environmental stress. Across all samples, community composition was dominated by archaeal and bacterial lineages commonly associated with geothermal ecosystems, particularly members of the phyla *Thermoproteota*, *Methanobacteriota*, *Deinococcota*, and *Actinomycetota*. These groups have been consistently reported as core components of terrestrial hot spring microbiomes and are widely recognized for their adaptation to extreme physicochemical conditions [9,13,17].

The high proportion of unclassified reads observed across most metagenomes is consistent with previous studies of geothermal and other extreme environments, where thermophilic microorganisms and uncultivated microbial lineages remain underrepresented in current reference databases [5,6,11]. This observation suggests that the El Tatio geothermal field harbors a substantial fraction of previously undescribed microbial diversity and further highlights the importance of genome-resolved metagenomics for expanding current genomic resources available for extremophilic microorganisms.

The predominance of *Thermoproteota* is consistent with their well-documented adaptation to high-temperature and low-organic-carbon environments, where sulfur metabolism and chemolithoautotrophy frequently support microbial growth [3,12]. Similarly, the detection of *Methanobacteriota* agrees with previous reports describing methanogenic archaea in El Tatio geothermal systems [14], supporting the notion that methanogenic processes may contribute to carbon cycling within these sediments.

Beyond these dominant taxa, the microbial assemblages contained a diverse low-abundance fraction composed of multiple archaeal and bacterial lineages. This so-called “rare biosphere” has been proposed as an important reservoir of phylogenetic and functional diversity in microbial ecosystems, particularly in environments characterized by strong environmental gradients and fluctuating physicochemical conditions [18,19]. Although the ecological significance of these low-abundance populations cannot be directly assessed from the present dataset, their occurrence expands the phylogenetic diversity detected within the El Tatio geothermal sediments.

Among the archaeal taxa detected at low abundance, the recurrent occurrence of *Promethearchaeota*, *Nanoarchaeota*, and *Woesearchaeota* across multiple samples is particularly noteworthy. These lineages are frequently associated with reduced genomes, specialized ecological strategies, and metabolic dependencies on partner organisms [20,21]. While such interactions cannot be inferred directly from metagenomic data alone, their repeated detection across geographically distinct geothermal microenvironments suggests that these groups represent stable components of the archaeal community.

The detection of *Promethearchaeota* is of particular interest given the evolutionary significance of *Asgard*-related archaea in current models of eukaryotic evolution [22,23]. Although present at low abundance, their consistent occurrence across multiple geothermal sites indicates that these deeply branching archaeal lineages are not restricted to isolated habitats but may be recurrent members of thermophilic microbial communities in extreme environments.

On the bacterial side, the identification of thermophilic phyla such as *Aquificota*, *Thermodesulfobacteriota*, *Thermotogota*, *Rhodothermia*, and *Deferribacterota* further highlights the phylogenetic diversity present within these geothermal sediments. Many representatives of these groups have previously been reported in geothermal springs and hydrothermal sediments worldwide, where they are associated with hydrogen oxidation, sulfur cycling, and anaerobic metabolic processes [3,12,15,16,24]. Their occurrence in El Tatio therefore reinforces the view that geothermal ecosystems support metabolically diverse bacterial communities adapted to extreme environmental conditions.

The coexistence of dominant thermophilic taxa and diverse low-abundance lineages highlights the phylogenetic complexity of the El Tatio geothermal microbiome. From a genome-resolved perspective, this diversity represents an important source of metabolic and enzymatic potential, providing the ecological context for the broad repertoire of hydrolase-encoding genes identified in the reconstructed genomes.

### 3.2. Genome-Resolved Insights into Thermophilic Adaptation and Functional Potential

Advances in genome-resolved metagenomics have transformed the study of microbial life in extreme environments by enabling the recovery of genomes from uncultivated microorganisms and linking taxonomic identity with functional potential [5,6]. In the present study, the reconstruction of 657 metagenome-assembled genomes (MAGs), including 273 high-quality and 190 near-complete genomes, substantially expands the genomic resources currently available for microorganisms inhabiting the El Tatio geothermal field. To our knowledge, this represents one of the most extensive genome-resolved surveys conducted in this high-altitude geothermal ecosystem.

Large-scale genome-resolved studies have demonstrated that geothermal environments harbor a considerable fraction of previously uncharacterized microbial diversity and metabolic potential [6,25]. The recovery of hundreds of medium- and high-quality MAGs from El Tatio therefore provides an important framework for investigating microbial adaptation to the combined effects of high temperature, reduced atmospheric pressure, and intense solar radiation characteristic of the Andean Altiplano.

The high completeness and low contamination observed for most reconstructed MAGs provide confidence for downstream taxonomic and functional analyses, enabling robust genome-resolved investigations of microbial diversity and metabolic potential within the El Tatio geothermal sediments.

The taxonomic composition of the recovered MAGs was consistent with observations from geothermal systems worldwide, including Yellowstone, Iceland, and Tibetan hot springs, where *Thermoproteota*, *Methanobacteriota*, *Deinococcota*, and *Actinomycetota* frequently dominate thermophilic communities [9,17]. The recurrence of these lineages across geographically distant hydrothermal environments suggests that similar environmental constraints may promote convergent patterns of community assembly and adaptation.

Beyond taxonomic characterization, the recovered MAG collection provides a genome-resolved framework for linking microbial lineages with specific metabolic functions. This genomic context proved particularly valuable for exploring the distribution of putative hydrolase-encoding genes and identifying candidate enzymes associated with thermophilic bacterial and archaeal taxa.

### 3.3. Functional and Biotechnological Implications of Thermophilic Enzyme Repertoires

The identification of 612 high-confidence putative hydrolase-encoding genes highlights the extensive hydrolytic potential present within the El Tatio geothermal microbiome. Although these candidates represent a highly filtered subset of the 878,934 initial hydrolase-like matches detected during similarity-based screening, their retention under stringent sequence identity and alignment-length thresholds provides increased confidence in their functional assignment. The diversity of recovered hydrolases suggests that thermophilic microbial communities inhabiting geothermal sediments possess a broad repertoire of enzymes involved in the degradation and transformation of complex organic substrates.

The enzyme classes identified in this study, including esterases, lipases, proteases, and glycoside hydrolases, are directly involved in the degradation of complex organic substrates and are among the most widely exploited enzyme groups in industrial biotechnology [1,26]. The predominance of esterases and lipases within the filtered dataset is particularly noteworthy given the increasing interest in thermostable hydrolases for applications requiring elevated temperatures, organic solvents, and other harsh processing conditions. In addition, enzymes derived from archaeal lineages are often characterized by enhanced structural robustness and catalytic performance under extreme physicochemical conditions, further reinforcing their potential biotechnological relevance [27].

Genome-resolved analyses revealed that hydrolase-associated sequences were unevenly distributed across the reconstructed MAGs, as evidenced by variation in hydrolase gene counts among reconstructed genomes (Table S2, columns 5 and 6), with a subset of MAGs encoding a large fraction of the detected enzymatic repertoire. This pattern suggests that hydrolytic potential may not be uniformly distributed across the community but instead concentrated within specific microbial lineages. However, differences in MAG completeness may also have contributed to the observed variation, because less complete MAGs may contain a smaller fraction of the original gene repertoire and therefore fewer detectable hydrolase-associated sequences. Accordingly, lineage-specific functional specialization and differences in genome recovery should be considered non-mutually exclusive explanations for the uneven gene counts. While biases associated with metagenomic assembly and genome binning cannot be fully excluded, the use of multiple binning algorithms (MaxBin2, MetaBAT2, and CONCOCT) followed by DAS Tool refinement supports the robustness of this observation. Similar patterns of functional specialization have been reported in microbial ecosystems where distinct taxa contribute disproportionately to metabolic processes, resulting in functional partitioning across microbial communities [28].

Although hydrolytic potential was concentrated in a subset of genomes, hydrolase-associated genes were detected across phylogenetically diverse bacterial and archaeal taxa, indicating that these functions are broadly distributed throughout the geothermal microbial community despite differences in genome content and completeness.

An important outcome of the genome-resolved approach was the ability to associate hydrolase candidates with specific bacterial and archaeal lineages. While the majority of hydrolase-rich MAGs were affiliated with thermophilic bacterial taxa such as *Meiothermus*, *Thermus*, *Calidithermus*, and *Caldilinea*, several candidates were also recovered from archaeal groups including *Thermoproteus*, *Sulfolobales*, *Pyrobaculum*, and members of the *Acidilobaceae*. The detection of hydrolase-associated genes within these archaeal genomes expands the phylogenetic breadth of potential enzyme sources identified in the El Tatio geothermal system and highlights the importance of considering archaeal diversity in future enzyme discovery efforts.

From a broader perspective, the genome-resolved linkage between hydrolase-encoding genes and specific microbial taxa provides an important framework for prioritizing candidate enzymes and understanding the ecological distribution of hydrolytic functions in geothermal ecosystems. These findings demonstrate how genome-resolved metagenomics can bridge microbial diversity and functional potential, providing a robust taxonomic framework for prioritizing candidate enzymes for future biochemical characterization and enzyme bioprospecting.

### 3.4. Bioprospecting Potential of High-Altitude Geothermal Microbiomes

Extreme environments have increasingly been recognized as reservoirs of enzymes with unusual catalytic properties. Thermophilic microorganisms in geothermal systems are subjected to strong selective pressures, including elevated temperatures, limited nutrient availability, and intense radiation exposure, which drive the evolution of proteins with enhanced structural stability and catalytic robustness. These characteristics make thermophilic enzymes particularly attractive for biotechnological applications [29,30].

The identification of 612 high-confidence putative hydrolase-encoding genes and the recovery of multiple candidates with predicted melting temperatures exceeding 80 °C further support the potential of the El Tatio geothermal microbiome as a source of thermophilic enzymes. Particularly noteworthy was the predominance of esterases and lipases, which exhibited the highest average predicted thermostability values, as well as individual candidates with predicted melting temperatures reaching 87.6 °C. Although these predictions require experimental validation, they are consistent with the thermal adaptations expected for proteins encoded by microorganisms inhabiting geothermal environments.

Comparative metagenomic studies conducted in geothermal systems worldwide have shown that thermophilic communities are frequently enriched in genes related to carbohydrate metabolism, protein degradation, and stress response pathways [1,17,24]. Advances in metagenomics-driven enzyme discovery have further expanded the identification of novel catalytic functions from extreme environments, enabling the exploration of previously uncharacterized enzymatic diversity with industrial potential [31]. The functional patterns observed in the El Tatio sediments are consistent with these findings and support the notion that high-temperature ecosystems converge toward similar functional profiles, particularly in pathways associated with organic matter degradation and adaptation to thermal stress, despite geographic separation.

The environmental conditions of the El Tatio geothermal field, including high altitude, low atmospheric pressure, and intense solar radiation, represent a combination of stressors that is uncommon among geothermal systems. These factors may contribute to the selection of microbial lineages and proteins with physiological and biochemical adaptations beyond thermotolerance alone. In this context, El Tatio provides a unique environmental framework for exploring enzyme systems shaped by multiple simultaneous stress conditions, potentially leading to the discovery of biocatalysts with distinctive stability profiles and functional properties.

### 3.5. Limitations and Future Directions for Genome-Resolved Enzyme Discovery

Genome-resolved metagenomics revealed that a substantial fraction of the microbial diversity present in El Tatio geothermal sediments corresponds to uncultivated and poorly characterized lineages, particularly within the archaeal domain. This pattern is consistent with previous studies showing that extreme environments harbor large proportions of microbial taxa lacking cultured representatives, thereby limiting functional characterization through traditional microbiological approaches [6,32]. In addition to dominant thermophilic archaeal groups, the detection of low-abundance and deeply branching lineages, including *Promethearchaeota* and other uncultivated taxa, highlights the phylogenetic complexity of these geothermal communities. Such lineages are frequently associated with specialized ecological niches and reduced or highly specialized metabolic repertoires [33,34]. However, because these organisms were inferred from metagenome-assembled genomes rather than cultured isolates, their ecological roles remain largely unresolved.

From a functional perspective, the detection of hydrolase-encoding genes within MAGs affiliated with uncultivated taxa suggests that these microorganisms possess the genomic potential to participate in organic matter transformation within geothermal sediments. Nevertheless, gene presence alone does not demonstrate transcription, translation, enzymatic activity, substrate specificity, or ecological contribution. Consequently, the functional roles inferred in this study should be interpreted as potential capabilities rather than confirmed biological activities. Integrating metagenomic data with transcriptomic, proteomic, and cultivation-based approaches will be necessary to better understand the ecological significance of these genes and their contribution to ecosystem functioning.

Several methodological limitations should also be considered. Hydrolase candidates were identified through metagenome-assembled genomes and sequence-based similarity searches; therefore, both functional and taxonomic assignments remain putative. Genome binning may be affected by incomplete genome recovery, contamination, strain-level heterogeneity, and potential misassignment of contigs, particularly for low-abundance populations. Although the use of multiple binning algorithms combined with DAS Tool refinement increases confidence in genome reconstruction, these limitations are inherent to genome-resolved metagenomic analyses.

Similarly, thermostability was assessed using PPTStab predictions rather than experimental melting-temperature measurements. While this approach provides an effective strategy for prioritizing candidates from large genomic datasets, predicted melting temperatures should be interpreted as screening indicators rather than validated biochemical properties. Future research should therefore focus on heterologous expression, biochemical characterization, and structural analyses to validate the catalytic activity, substrate specificity, and thermal stability of the most promising hydrolase candidates identified in this work.

Despite these limitations, proteins encoded by uncultivated or poorly characterized microorganisms may substantially expand the sequence space available for enzyme discovery, particularly because many remain underrepresented in current reference databases and may possess structural or functional adaptations associated with extreme physicochemical conditions [1,35]. Integrating genome-resolved metagenomics with transcriptomic, proteomic, and structure-based approaches will improve the functional interpretation of candidate enzymes and facilitate the identification of proteins with desirable biotechnological properties [36]. In addition, the combination of machine learning-guided enzyme mining, protein structure prediction, and large-scale environmental sequencing represents a promising framework for accelerating enzyme discovery from extreme environments. Together, these approaches may help bridge the gap between genomic prediction and experimental validation, reinforcing the value of high-altitude geothermal ecosystems as reservoirs of microbial and functional diversity for future bioprospecting efforts.

## 4. Methods and Materials

**Figure 4:**
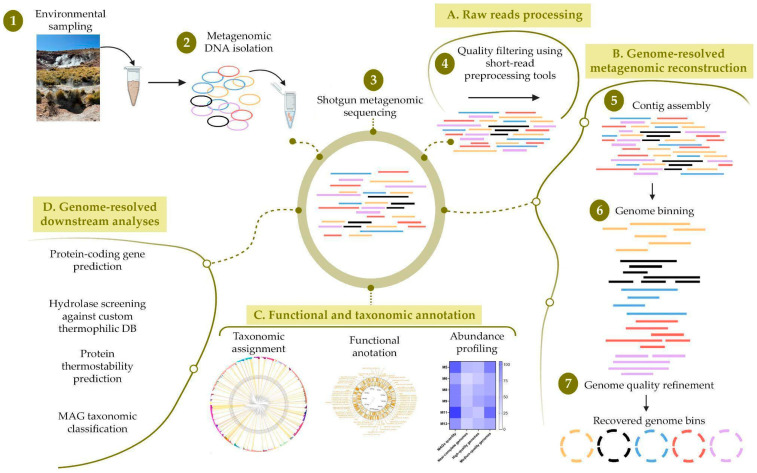
Overview of the genome-resolved metagenomic workflow applied to geothermal sediment samples. (**A**) Environmental sampling, metagenomic DNA extraction, and shotgun sequencing followed by raw read preprocessing and quality filtering. (**B**) Reconstruction of metagenome-assembled genomes through contig assembly, genome binning, and genome quality refinement. (**C**) Functional and taxonomic annotation, including taxonomic assignment, functional gene annotation and abundance profiling. (**D**) Genome-resolved downstream analyses based on protein-coding gene prediction, screening of hydrolase sequences against a custom thermophilic enzyme database, protein thermostability prediction, and taxonomic classification of MAGs.

The overall analytical workflow applied in this study is summarized in Figure 4.

### 4.1. Study Area and Sample Collection

Six geothermal sediment and microbial mat samples (M5, M6, M8, M9, M11, and M13) were collected from the El Tatio geothermal field, located in the Antofagasta Region of northern Chile within the Andean Altiplano, at approximately 4300 m above sea level (22°22′00″ S, 67°59′23″ W). El Tatio is an active hydrothermal system characterized by geysers, hot springs, geothermal ponds, and bubbling mud pools, where elevated temperatures, variable pH, and heterogeneous geochemical gradients create diverse microhabitats for thermophilic microorganisms.

Sampling was conducted in areas exhibiting active surface geothermal manifestations. Sites were selected based on in situ temperature measurements ranging from 70 to 82 °C, recorded using an infrared thermometer (UT309C, UNI-T, Dongguan, Guangdong, China)). Surface sediments and microbial mat material were aseptically collected using sterile spatulas and transferred to sterile conical tubes for transport to the Laboratory of Extremophilic Microorganisms at the Instituto Antofagasta, Universidad de Antofagasta. Upon arrival at the laboratory, samples were subdivided and stored at −80 °C and 4 °C until further processing.

### 4.2. DNA Extraction and Quality Assessment

Genomic DNA was extracted from geothermal sediment and microbial mat samples using the ZymoBIOMICS™ DNA Miniprep Kit (Zymo Research, Irvine, CA, USA) according to the manufacturer’s instructions. Mechanical cell disruption was performed by bead beating to facilitate lysis of microbial cells embedded within the environmental matrix. Purified DNA was eluted in nuclease-free water and stored at −20 °C until further processing.

DNA concentration was quantified using the Qubit™ 1X dsDNA Assay Kit and a Qubit™ fluorometer (Invitrogen, Thermo Fisher Scientific, Waltham, MA, USA). DNA integrity was assessed by electrophoresis on 0.7% (*w*/*v*) agarose gels prepared in 1× TAE buffer. Only high-quality DNA extracts were used for metagenomic library preparation and sequencing.

### 4.3. Shotgun Metagenomic Sequencing and Quality Control

Purified environmental DNA was sequenced by Macrogen Inc. (Seoul, Republic of Korea) using the Illumina NovaSeq X platform (Illumina Inc., San Diego, CA, USA) with TruSeq Nano DNA library preparation (META). Sequencing generated paired-end reads (150 bp × 2) with an approximate sequencing depth of 12–13 Gbp per sample. Raw sequencing output was delivered as paired FASTQ files and used for downstream bioinformatic analyses, including quality filtering, taxonomic profiling, metagenomic assembly, genome reconstruction, and functional annotation.

### 4.4. Read Quality Control

Raw metagenomic reads were processed using fastp v0.23.2 (HaploX Biotechnology, Shenzhen, China) [37] to remove adapter sequences, trim low-quality bases, and discard low-quality or excessively short reads. This preprocessing step was performed to minimize sequencing artifacts and improve the reliability of downstream analyses. Quality-filtered reads were subsequently used for taxonomic classification, metagenomic assembly, genome reconstruction, and functional annotation.

### 4.5. Taxonomic Profiling of Metagenomic Reads

Taxonomic classification of quality-filtered reads was performed using Kraken2 v2.1.3 (Johns Hopkins University, Baltimore, MD, USA) [38] with the Standard-8 reference database. Kraken2 assigns taxonomic labels to sequencing reads through exact k-mer matching against a comprehensive reference genome collection, enabling rapid and sensitive taxonomic profiling of complex metagenomic datasets.

Taxonomic assignments were summarized at multiple taxonomic ranks, including phylum, family, and genus levels, to characterize archaeal and bacterial community composition across the geothermal sediment and microbial mat samples. Relative abundances were subsequently used to compare community structure among sampling sites and to identify dominant and low-abundance microbial lineages.

### 4.6. Metagenomic Assembly

Quality-filtered reads were assembled using MEGAHIT v1.2.9 (Shenzhen Institutes of Advanced Technology, Chinese Academy of Sciences, Shenzhen, China) [39], a de Bruijn graph-based assembler specifically designed for large and complex metagenomic datasets. Assembly quality was evaluated using standard assembly metrics, including total assembly size, contig length distribution, N50, and L50 values.

To improve the reliability of downstream genome-resolved analyses, contigs shorter than 1 kb were excluded prior to genome binning and functional annotation. Short contigs often contain insufficient compositional and coverage information for accurate binning and may contribute disproportionately to assembly fragmentation and taxonomic ambiguity. Consequently, only contigs ≥ 1 kb were retained for subsequent genome reconstruction and comparative analyses.

### 4.7. Gene Prediction

Protein-coding genes were predicted from assembled contigs using Prodigal v2.6.3 (Oak Ridge National Laboratory, Oak Ridge, TN, USA) [40] in metagenomic mode, which is specifically optimized for fragmented and heterogeneous metagenomic assemblies. Predicted open reading frames (ORFs) were translated into protein sequences and subsequently used for downstream analyses, including similarity-based screening against the thermophile-derived hydrolase reference database, thermostability prediction, and functional annotation of selected candidates.

### 4.8. Construction of a Thermophile-Derived Hydrolase Reference Database

A custom thermophile-derived hydrolase reference database was constructed as a targeted screening resource to facilitate the identification of putative hydrolase-encoding genes within the metagenomic datasets. The database was designed as an exploratory similarity-based screening framework rather than as a definitive functional annotation resource.

Protein sequences were retrieved from the NCBI Protein database [41] using combinations of thermophile-associated taxonomic terms and hydrolase-related functional keywords. Taxonomic queries focused on microbial genera previously reported to include thermophilic or hyperthermophilic representatives, including *Thermus*, *Hyperthermus*, *Thermophilum*, *Thermobacillus*, *Thermococcus*, *Geobacillus*, *Parageobacillus*, *Methanopyrus*, *Pyrococcus*, *Pyrolobus*, and *Thermotoga*. The source organisms represented in the final database are provided in Table S4.

Protein records were retained when their NCBI annotations contained hydrolase-related descriptions corresponding to enzyme groups of interest, including lipases, esterases, proteases, pectinases, glycoside hydrolases, and related derivative annotations. Broader hydrolase-associated descriptions, such as “hydrolase”, “alpha/beta hydrolase”, “glycoside hydrolase”, and “HAD family hydrolase”, were also included. Records annotated exclusively as “hypothetical protein”, “uncharacterized protein”, or lacking a hydrolase-related functional description were excluded from the database.

The final database comprised 3392 protein sequences representing diverse hydrolase families associated with thermophilic microorganisms. For methodological transparency, annotation descriptions were standardized and grouped into broad functional categories, including generic hydrolases, protease/peptidase-related enzymes, esterase/carboxylesterase-related enzymes, polysaccharide-degrading hydrolases, and lipase/phospholipase-related enzymes. The distribution of these categories is summarized in Table S5.

Because the database was developed as a screening resource, functional assignments derived from sequence similarity were interpreted conservatively. To reduce potential annotation bias, selected high-confidence candidates were subsequently evaluated using BLASTp searches against the NCBI clustered non-redundant protein database and KEGG Ortholog annotation through KOfamKO analyses.

### 4.9. Genome Binning and Reconstruction of Metagenome-Assembled Genomes (MAGs)

Metagenome-assembled genomes (MAGs) were reconstructed from assembled contigs using the MetaWRAP pipeline (University of Chicago, Chicago, IL, USA) [42]. Genome binning was performed using three complementary algorithms integrated within the pipeline: MaxBin2 v2.2.7 (University of Alberta, Edmonton, AB, Canada) [43], MetaBAT2 (Lawrence Berkeley National Laboratory, Berkeley, CA, USA) [44] and CONCOCT (Chalmers University of Technology, Gothenburg, Sweden) [45]. These tools employ different strategies based on sequence composition, coverage patterns, and probabilistic clustering, allowing recovery of genome bins with complementary strengths and reducing biases associated with individual binning approaches.

The resulting genome bins were subsequently consolidated using DAS Tool v1.1.7 (University of Queensland, Brisbane, Australia) [46], which integrates predictions from multiple binning algorithms to generate a non-redundant set of optimized genome bins. The refined bins were retained for downstream quality assessment, taxonomic classification, and functional analyses.

### 4.10. MAG Quality Assessment

The quality of reconstructed MAGs was evaluated using CheckM v1.2.3 (University of Queensland, Brisbane, Australia), which estimates genome completeness and contamination based on lineage-specific single-copy marker genes. Genome quality was assessed using completeness and contamination-based categories adapted from the MIMAG framework [47]. Because the present study evaluated CheckM completeness and contamination, the groups used here were treated as operational, nested quality thresholds rather than formal MIMAG classifications. Genomes with 100% completeness were reported as such; genomes with ≥95% completeness and <5% contamination were classified as near-complete genomes, genomes with ≥90% completeness and <5% contamination were classified as high-quality draft genomes, whereas genomes with ≥50% completeness and <10% contamination were classified as medium-quality genomes. MAGs meeting these quality thresholds were classified accordingly and prioritized for downstream genome-resolved analyses.

### 4.11. Identification Pipeline for Putative Hydrolase-Encoding Genes

Predicted protein sequences derived from assembled contigs were compared against the curated thermophile-derived hydrolase reference database using DIAMOND v2.1.8 (Max Planck Institute for Biology Tübingen, Tübingen, Germany) in blastp mode (National Center for Biotechnology Information (NCBI), Bethesda, MD, USA) [48,49]. Sequence similarity searches were performed to identify proteins homologous to known hydrolase families represented in the custom reference dataset.

DIAMOND matches were filtered using a minimum amino-acid identity threshold of ≥60%, an alignment length of ≥250 amino acids, and an e-value threshold of 1 × 10^−5^. The identity threshold was selected to retain candidates showing substantial similarity to previously characterized thermophile-derived hydrolases, thereby supporting putative enzyme-family assignments while reducing weak or ambiguous matches. The alignment-length threshold was applied to exclude short ORFs, fragmented proteins, and partial alignments restricted to conserved domains or catalytic motifs, which may otherwise inflate functional predictions in metagenomic datasets. Collectively, these criteria were designed to prioritize specificity over sensitivity during candidate selection, acknowledging that highly divergent hydrolases or naturally shorter enzymes may have been underrepresented.

When multiple matches were identified for the same query sequence, only the highest-scoring hit based on bit score was retained. A more stringent identity threshold of ≥90% was subsequently applied to prioritize highly similar candidates for detailed functional annotation and thermostability prediction.

Hydrolase candidates were defined as sequences matching curated reference proteins associated with hydrolase activity, including esterases, lipases, proteases, and polysaccharide-degrading enzymes. Functional assignments were initially inferred from sequence similarity and associated EC numbers when available.

To improve annotation confidence, selected high-confidence candidates, particularly those exhibiting elevated predicted melting temperatures, were further analyzed using BLASTp searches against the NCBI clustered non-redundant protein database and KEGG Ortholog annotation through KOfamKO analyses (Kyoto University, Kyoto, Japan) [50].

### 4.12. Prediction of Enzyme Thermostability

Hydrolase candidates identified through DIAMOND similarity searches were subjected to thermostability analysis using PPTStab (Department of Computational Biology, Indraprastha Institute of Information Technology Delhi, New Delhi, India) [51]. Sequences meeting the general filtering criteria (≥60% amino-acid identity and ≥250 amino acids in alignment length) were selected to estimate the thermal stability profiles of putative hydrolases recovered from the metagenomic datasets.

Thermostability predictions were performed using the standalone version of PPTStab, a machine learning-based tool that estimates protein melting temperature (Tm) from amino acid sequence features. The model infers thermostability using sequence-derived physicochemical descriptors trained on datasets of experimentally characterized proteins. Analyses were conducted in batch mode using default parameters.

Estimated melting temperatures were used to compare the relative thermal stability of candidate enzymes and to prioritize hydrolases for downstream analyses. These values should be interpreted as computational estimates of thermostability rather than experimentally validated melting temperatures.

### 4.13. Data Availability and Reproducibility

All bioinformatic analyses were performed using publicly available software and reproducible computational workflows. Sequencing datasets and reconstructed metagenome-assembled genomes will be deposited in public repositories upon manuscript acceptance in accordance with journal data-sharing policies and FAIR data principles.

## 5. Conclusions

This study provides a genome-resolved characterization of thermophilic microbial communities inhabiting geothermal sediments of the El Tatio geothermal field, one of the largest high-altitude hydrothermal systems in the Andean Altiplano. The integration of shotgun metagenomics, genome reconstruction, and functional annotation enabled the recovery of 657 metagenome-assembled genomes (MAGs), including 273 high-quality and 190 near-complete genomes, substantially expanding the genomic resources currently available for microorganisms associated with this geothermal ecosystem. These findings further support the growing importance of genome-resolved approaches for exploring microbial diversity and functional potential in environments where most microorganisms remain uncultivated [4,36].

Functional screening identified 612 high-confidence putative hydrolase-encoding genes spanning multiple enzyme classes, including esterases, lipases, proteases, and glycoside hydrolases. Genome-resolved analyses demonstrated that these genes are distributed across diverse bacterial and archaeal lineages, linking hydrolytic potential to specific thermophilic taxa and providing a framework for prioritizing candidate enzymes for future investigation. The recovery of hydrolase candidates within archaeal MAGs extends previous genome-resolved studies of geothermal microbiomes by expanding the catalog of putative thermostable enzymes associated with underexplored archaeal lineages, highlighting these microorganisms as promising targets for future enzyme discovery and biotechnological applications [27].

Predicted thermostability analyses revealed numerous candidates with thermal stability profiles consistent with adaptation to geothermal environments, including enzymes with estimated melting temperatures exceeding 80 °C and a maximum predicted value of 87.6 °C. Although experimental validation remains necessary, these results are consistent with previous studies highlighting the industrial relevance of thermostable enzymes and the value of geothermal ecosystems as reservoirs of biocatalytic diversity [2,52].

The unique environmental conditions of the Andean Altiplano, where geothermal activity is combined with high altitude, intense solar radiation, and pronounced physicochemical gradients, reinforce the value of El Tatio as a natural laboratory for studying microbial adaptation and enzyme evolution under multiple environmental stressors. Overall, this work demonstrates how genome-resolved metagenomics can connect microbial diversity with functional potential, providing a robust foundation for future enzyme validation, structural characterization, and bioprospecting efforts in extreme environments.

## Figures and Tables

**Table 1:** In situ temperature of geothermal sediment and microbial mat samples.

Sample ID	Temperature (°C)	pH	Sample Type
M5	78	6.69	Sediment
M6	82	ND	Sediment
M8	70	5.78	Microbial mat
M9	70	6.79	Sediment
M11	75	3.80	Sediment
M13	80	ND	Microbial mat

ND: not determined.

**Table 2:** DNA concentration obtained from geothermal samples.

Sample ID	DNA Quantity (ng/µL)	Total DNA Recovered (ng)
M5	7.62	762
M6	9.29	929
M8	5.41	541
M9	2.53	253
M11	1.29	129
M13	9.44	944

**Table 3:** Summary of metagenomic sequencing output.

Sample ID	Library Concentration	Total Bases (bp)	Total Reads	GC Content (%)	AT Content (%)
M5	31.23	12.860.713.186	85.170.286	57.2	42.8
M6	32.63	12.098.497.802	80.122.502	53.1	46.9
M8	31.20	12.032.015.220	79.682.220	57.7	42.3
M9	33.66	12.469.112.202	82.576.902	58.5	41.5
M11	39.77	12.352.082.068	81.801.868	56.8	43.2
M13	35.91	12.891.685.400	85.375.400	60.5	39.5

**Table 4:** Summary of metagenomic read classification using Kraken2.

Sample ID	Classified Reads (%)	Unclassified Reads (%)
M5	29.43	70.57
M6	63.79	36.21
M8	24.56	75.44
M9	25.61	74.39
M11	36.90	63.10
M13	21.70	78.30

**Table 5:** Summary of metagenomic assembly metrics, gene prediction, and preliminary hydrolase-like sequence identification.

Sample ID	Number of Contigs (>1000 bp)	N50	L50	Predicted ORFs	Hydrolase-Like Matches (Unfiltered)
M5	74,405 contigs (>1000 bp)	15,404	4055	708,600	148,693
M6	43,207 contigs (>1000 bp)	9133	3600	408,018	78,402
M8	91,881 contigs (>1000 bp)	4424	11,662	870,826	152,328
M9	78,250 contigs (>1000 bp)	6891	6046	739,816	147,710
M11	99,152 contigs (>1000 bp)	5733	10,517	970,453	175,617
M13	110,121 contigs (>1000 bp)	5414	12,988	943,151	176,184

**Table 6:** Mean properties of predicted hydrolase classes across the six geothermal sediment samples (± SD).

Enzyme Class	Mean Number of Sequences per Sample	Mean Sequence Length (aa)	Mean Predicted Tm (°C)	Predicted Tm Range (°C)
Lipases	55 ± 2.80	322 ± 1.17	79.89 ± 0.71	48.88–82.95
Esterases	55 ± 3.99	406 ± 31.44	81.07 ± 0.31	57.73–85.65
Proteases	19 ± 6.12	515 ± 51.84	74.93 ± 0.93	56.62–83.32
Pectinases	2 ± 0.58	496 ± 36.37	53.82 ± 6.18	47.97–62.78
Other hydrolases	86 ± 34.31	404 ± 15.91	79.76 ± 1.11	45.27–86.64

**Table 7:** Distribution of MAG quality categories across samples.

Sample ID	MAGs Quantity	Complete Genomes (100% Completeness, < 5% Contamination)	Near-Complete MAGs (≥95% Completeness, <5% Contamination)	High-Quality MAGs (≥ 90% Completeness, <5% Contamination)	Medium-Quality MAGs (≥50% Completeness, <10% Contamination)
M5	124	1	39	58	84
M6	57	3	22	31	48
M8	124	2	29	42	67
M9	101	3	33	49	74
M11	131	1	39	54	90
M13	120	-	28	39	67
Total	657	10	190	273	430

**Table 8:** Selected metagenome-assembled genomes (MAGs) and associated hydrolase candidates identified from geothermal sediments of the El Tatio geothermal field.

MAG ID	Closest GTDB Taxon	EC	Putative Function (Based on Custom Database Match)	Protein Length (aa)	Predicted Tm (°C)
M5 120ccT	*Meiothermus*	3.7.1.9	2-hydroxymuconic semialdehyde hydrolase	265	82.7
		3.2.1.-	glycoside hydrolase family 13 protein	514	82.1
		3.2.1.-	glycoside hydrolase family 13 protein	563	77.9
		3.2.1.-	glycoside hydrolase family 125 protein	558	77.2
		3.4.24.-	protease TldD	464	76.6
M6 43ccT	*Thermus antranikianii*	3.5.-.-	carbon-nitrogen hydrolase	283	87.6
		3.-.-.-	Zn-dependent hydrolase	285	85.1
		3.-.-.-	alpha/beta fold hydrolase	312	85.0
		3.4.21.-	serine protease	282	84.9
		3.2.1.-	glycoside hydrolase family 36 protein	476	84.5
M8 39mxB	*Calidithermus chliarophilus*	3.2.1.3	glycoside hydrolase family 15 protein	762	82.4
		3.2.1.-	glycoside hydrolase family 13 protein	510	81.0
		3.2.1.-	glycoside hydrolase family 125 protein	583	79.3
		3.4.21.-	serine protease	420	78.6
		3.2.1.-	glycoside hydrolase family 31 protein	788	78.4
M9 66ccT	*Meiothermus*	3.2.1.3	glycoside hydrolase family 15 protein	255	79.5
		3.5.5.-	nitrilase-related carbon-nitrogen hydrolase	287	78.0
		3.2.1.1	alpha-amylase family glycosyl hydrolase	531	71.5
		3.4.21.92	Clp protease	730	71.4
		3.4.21.53	Lon protease	792	69.8
M11 29mtB	*Meiothermus_B silvanus*	3.5.-.-	carbon-nitrogen hydrolase	283	87.6
		3.4.21.-	serine protease	258	81.0
		3.2.1.3	glycoside hydrolase family 15 protein	763	80.4
		3.4.-.-	ATP-dependent protease	494	78.4
		3.2.1.-	glycoside hydrolase family 13 protein	516	78.3
M13 148ccT	*Caldilinea*	3.1.2.1	Acyl-CoA hydrolase	418	62.5
		3.4.21.-	serine protease	267	61.4
M6 12ccT	*Thermoproteus*	3.4.-.-	ATP-dependent protease	461	64.2
		3.4.-.-	ATP-dependent protease	465	64.2
M11 39mtB	*Thermoproteus*	3.4.-.-	ATP-dependent protease	734	63.9
M11 116mtB	*Pyrobaculum aerophilum*	3.4.-.-	ATP-dependent protease	436	64.5
		3.4.-.-	ATP-dependent protease	444	62.2
M6 52mtB	*Sulfolobales*	3.13.2.1	S-adenosyl-L-homocysteine hydrolase	413	79.7
		3.4.-.-	ATP-dependent protease	685	66.7
		3.4.-.-	ATP-dependent protease	721	62.2
M11 26mtB	*Acidilobaceae*	3.4.-.-	ATP-dependent protease	726	62.2
M11 9ccT	*Sulfolobales*	3.13.2.1	S-adenosyl-L-homocysteine hydrolase	413	79.4
		3.4.-.-	ATP-dependent protease	302	67.8
		3.4.-.-	ATP-dependent protease	368	65.5

## Data Availability

The metagenomic sequencing data generated in this study have been deposited in the NCBI Sequence Read Archive (SRA) under BioProject accession PRJNA1503071. Additional data supporting the findings of this study are available within the article and its Supplementary Materials.

## Supplementary Materials

The following supporting information can be downloaded at: https://www.mdpi.com/article/10.3390/ijms27156905/s1.
