# Supplementary material for: Genome-Resolved Metagenomics Reveals Thermophilic Microbial Diversity and Putative Hydrolase-Encoding Genes in the El Tatio Geothermal Field
Source: Int J Mol Sci. 2026 Aug 1;27(15):6905. doi: 10.3390/ijms27156905 (PMC13467546; doi:10.3390/ijms27156905)
Supplement: Supplementary file 1 [file ijms-27-06905-s001.zip › ijms-4461864-supplementary.pdf]

Appendix A (Supplementary Data)

Table S1. Near-complete metagenome-assembled genomes (MAGs) reconstructed from geothermal microbial communities across samples M5–M13, showing genome quality metrics (completeness and contamination) and closest taxonomic affiliation according to GTDB-Tk.

**Sample ID**	**Near complete genomes**	**Completeness**	**Contamination**	**Closest GTDB-Tk classification**
M5	162ccT	97.3	2.73	f__J093
	155ccT	96.37	3.99	g__Meiothermus
	152ccT	95.83	0.93	g__GBS-DC
	151ccT	96.59	0	g__UBA2421
	150ccT	96.34	0.55	f__UBA2268
	142ccT	99.52	1.45	f__Syntrophobacteraceae
	126ccT	95.93	3.49	g__Gemmata
	120ccT	98.72	2.99	g__Meiothermus
	119ccT	98.54	2.29	g__Pelomicrobium
	110ccT	97.19	1.98	f__DSGV01
	99ccT	95.6	0	s__32-111
	95ccT	96.7	3.96	s__Tepidiforma
	94ccT	99.69	0	s__Chloroflexus
	89ccT	99.69	0	g__Roseiflexus
	79ccT	96.92	0.29	s__Calidifontimicrobium
	67ccT	100.0	1.75	g__Fervidobacterium

57ccT	97.27	0.11	f__B-17BO
32ccT	98.37	1.63	d__Bacteria
27ccT	96.77	0.97	s__SpSt-501
6ccT	97.46	0.48	f__B-17BO
96mtB	99.4	1.25	g__UBA2336
89mtB	98.71	0.81	g__Desulfosoma
85mtB	99.28	0.24	s__Fischerella
59mtB	95.48	1.29	f__JdFR-97
55mtB	95.86	2.53	g__Raineya
43mtB	99.39	0	s__UBA11096
18mtB	97.3	2.06	g__Limisphaera
7mtB	97.3	2.03	g__DSWX01
4mxB	99.52	0.0	g__PALSA-612
5mxB	96.76	4.55	s__UBA8783
19mxB	99.52	2.38	g__JAAYUG01
26mxB	96.3	4.63	f__Nitrospiraceae
27mxB	99.13	1.0	g__JANXAZ01
31mxB	98.76	0.0	g__Elioraea
35mxB	98.86	3.41	g__DSNY01
39mxB	99.38	2.88	s__UBA2250

	51mxB	97.8	0.0	g__RPQV01
	52mxB	98.42	3.32	g__SXOA01
M6	83ccT	99.06	0.94	s__O-77 sp013177315
	66ccT	98.07	1.69	s__Fischerella thermalis
	64ccT	98.57	0.48	f__B-17BO
	52ccT	96.19	3.39	g__Thermus_A
	43ccT	98.31	4.66	s__Thermus antranikianii
	36ccT	97.86	3.42	s__Calidithermus chliarophilus
	35ccT	98.81	1.79	g__Sulfurisphaera
	33ccT	100	1.65	g__DSNY01
	26ccT	98.54	1.81	g__Pelomicrobium
	20ccT	96.45	0.55	o__Kapabacteriales
	12ccT	100	0.37	g__Thermoproteus
	10ccT	95.94	3.63	f__B-17BO
	7ccT	99.69	0	g__Pelomicrobium
	6ccT	95.23	1.14	s__Thermogemmata fonticola
	3ccT	96.7	0.99	s__Tepidiforma bonchosmolovskayae
	2ccT	96.3	1.03	f__HRBIN12
	57mtB	100	0.94	o__Thermomicrobiales

	53mtB	95.3	1.71	g__OLB17
	52mtB	95.5	0	g__AG1
	34mtB	98.37	2.44	s__UBA11096 sp003534055
	20mtB	95.59	2.94	g__DSFO01
	1mxB	99.01	0.49	s__Thermoflavifilum aggregans
M8	157ccT	98.88	1.12	g__XYB12-FULL-38-5
	150ccT	96	2.36	s__Thermomonas hydrothermalis
	149ccT	95.6	0.55	s_32-111 sp002441125
	134ccT	95.62	0.2	s_Hydrogenophilus thermoluteolus
	114ccT	96.1	0.16	s_Tibeticola sp013693755
	106ccT	99.15	2.56	g_Holophaga
	105ccT	97.66	0.73	g_Hylemonella
	87ccT	98.11	0.07	s_Erythrobacter cryptus
	81ccT	98.57	3.1	g_Ch61
	77ccT	96.99	0.55	f__Kapabacteriaceae
	69ccT	99.62	1.15	s__Thermoanaerobaculum aquaticum
	56ccT	97.61	0.47	s__Tepidimonas thermarum
	30ccT	96.45	0.55	g__UBA4820

	18ccT	99.84	2.1	f__Syntrophobacteraceae
	3ccT	98.65	3.03	g__DSWX01
	64mtB	95.42	2.5	s__EMTCatB1 sp001950395
	52mtB	97.7	0	s__JAJTBL01 sp021286925
	29mtB	98.4	0.77	g__UBA6016
	18mtB	97.73	0.2	g__Desulfomonile
	14mtB	99.19	1.22	s__UBA11096 sp012517235
	12mtB	98.39	0.97	g__Calidifontimicrobium
	4mxB	99.15	2.47	g__Ignavibacterium
	6mxB	100.0	1.75	g__Ignavibacterium
	11mxB	98.17	2.66	s__JAAYVI01 sp012517235
	15mxB	98.37	3.86	f__DTGW01
	17mxB	99.16	0.56	g__JACPVF01
	28mxB	99.52	1.76	g__UBA8783
	30mxB	96.63	1.12	s__Thermus antranikianii
	39mxB	100.0	0.5	g__Meiothermus
M9	141ccT	98.29	0	g__JACYLR01
	138ccT	99.52	0	g__Chlorogloeopsis
	123ccT	98.72	4.7	g__Meiothermus

118ccT	95.23	1.14	s__Thermogemmata fonticola
107ccT	96.58	1.71	g__OLB17
95ccT	96.64	0.42	f__Beijerinckiaceae
94ccT	98.68	3.3	g__DTNP01
88ccT	98.68	1.98	f__SXQI01
75ccT	95.37	0.93	o__DTGP01
73ccT	98.71	0.81	g__Desulfosoma
67ccT	96.77	1.29	f__JdFR-97
62ccT	95.91	2.3	g__ELB16-189
54ccT	97.27	4.73	s__Thermoanaerobaculum aquaticum
40ccT	98.77	0.49	s__Thermoflavifilum aggregans
38ccT	96.45	0.55	f__NICIL-2
31ccT	99.19	0.81	s__Erythrobacter cryptus
20ccT	100	0	s__Thermogutta terrifontis
17ccT	99.32	4.08	g__Limisphaera
7ccT	96.55	1.15	f__JdFR-97
3ccT	96.7	4.4	g__DTLA01
29mtB	98.65	2.7	g__Pelomicrobium
3mxB	100.0	1.75	g__Caldilinea

	8mxB	99.69	0.94	g__Desulfosoma
	10mxB	97.97	2.03	g__Meiothermus
	14mxB	97.8	0.0	g__Sumerlaea
	19mxB	99.13	0.9	g__SXOA01
	23mxB	98.06	0.0	g__HRBIN30
	35mxB	96.7	1.21	f__UBA2268
	37mxB	99.53	0.94	f__Nap2-2B
	41mxB	99.09	4.55	f__UBA9159
	43mxB	100.0	0.91	g__JANXAZ01
	45mxB	97.09	0.97	g__RPQV01
	48mxB	95.25	3.1	g__Bellilinea
M11	186ccT	98.43	3.42	o__Gp7-AA8
	153ccT	97.81	2.03	f__SXQI01
	152ccT	99.69	1.65	g__DSNY01
	126ccT	95.3	3.42	s__Thermoanaerobaculum aquaticum
	102ccT	95.95	3.72	s__Chloroflexus aurantiacus
	89ccT	99.44	0	s__Rhodothermus bifroesti
	78ccT	98.65	3.25	s__Tepidiforma bonchosmolovskayae
	75ccT	98.5	0.43	g__Meiothermus

69ccT	99.69	0	g__SHND01
64ccT	97.97	1.63	g__JADGGM01
56ccT	96.86	3.15	g__GBS-DC
52ccT	95.73	1.71	f__HRBIN12
123mtB	96.2	2.73	g__DSWX01
116mtB	97.79	0.74	s__Pyrobaculum aerophilum
115mtB	98.06	0	g__Caldarchaeum
112mtB	98.39	0	o__ARK-15
109mtB	97.8	4.4	g__JABWBJ01
101mtB	96.21	0	g__HRBIN16
100mtB	98.03	0.1	g__Raineya
94mtB	97.3	2.73	g__UBA2336
92mtB	95.7	1.1	g__Pelomicrobium
91mtB	98.81	1.4	f__CSP1-4
61mtB	96.45	1.64	f__NICIL-2
43mtB	96.58	1.71	g__OLB17
29mtB	95.49	1.5	f__UBA12049
27mtB	100	0.91	g__JACRFR01
26mtB	95.89	0.63	g__DSZV01
9mtB	96.62	0.62	g__PCC-10605

	1mxB	95.73	0.41	g__SXOA01
	12mxB	99.52	0.56	g__OLB14
	14mxB	99.51	0.99	g__DSGC01
	20mxB	99.51	0.49	g__Fen-1038
	23mxB	98.13	1.87	g__Thermomicrobium
	24mxB	97.47	1.68	s__Thermoflavifilum sp014961315
	30mxB	97.3	3.38	g__DTLC01
	39mxB	99.01	4.31	g__VBAS01
	46mxB	99.09	0.91	g__JANWVO01
	52mxB	99.09	1.09	g__UBA2250
	56mxB	97.97	1.1	s__UBA11096 sp003534055
M13	172ccT	99.13	0.91	g__JANXAZ01
	168ccT	97.51	2.05	f__SG8-40
	160ccT	99.52	0.24	g__Chlorogloeopsis
	148ccT	99.09	3.64	g__Caldilinea
	143ccT	99.38	1.85	g__DSNY01
	141ccT	96.96	0.91	f__Sphingomonadaceae
	97ccT	95.7	4.84	o__MHYJ01
	92ccT	98.65	4.05	o__Pedosphaerales
	79ccT	98.54	2.29	g__Pelomicrobium

70ccT	96.7	2.75	g__JADGGM01
50ccT	99.53	2.36	s__JACYLU01 sp015272325
48ccT	96.29	0.75	g__Thermogemmata
42ccT	96.48	3.26	g__Chloracidobacterium
41ccT	95.73	1.71	g__JANWVO01
34ccT	96.76	4.55	f__Gaiellaceae
25ccT	95.4	0	f__NICIL-2
21ccT	96.59	1.14	f__UBA1924
20ccT	97.59	2.08	g__Thermogutta
10ccT	96.45	1.64	f__J093
96mtB	96.86	0	g__Raineya
79mtB	98.18	0.91	g__JALHNB01
68mtB	97.27	0.91	g__CF-162
41mtB	95.23	1.14	g__OLB17
17mtB	95.53	1.36	s__UBA11096 sp003534055
15mtB	96.86	1.89	s__Chloroflexus aurantiacus
10mxB	95.96	3.64	g__OMJK01
11mxB	99.4	1.25	g__DSGC01
13mxB	96.21	4.44	g__Meiothermus

Table S2. Completeness, contamination, and hydrolase gene repertoires of every MAG over 50% completeness and less than 10% contamination.

Sample ID	MAG ID	Completeness (%)	Contamination (%)	Predicted ORFs	Hydrolase genes matches
M5	172ccT	99.37	6.18	2631	724
	162ccT	97.3	2.73	3180	840
	157ccT	79.91	7.86	1584	243
	155ccT	96.37	3.99	2787	1593
	152ccT	95.83	0.93	2409	708
	151ccT	96.59	0	3022	884

150ccT	96.34	0.55	2625	657
143ccT	82.01	2.32	2453	604
142ccT	99.52	1.45	4338	1260
126ccT	95.93	3.49	4352	909
120ccT	98.72	2.99	3373	2040
119ccT	98.54	2.29	3454	901
110ccT	97.19	1.98	3804	1494
109ccT	56.38	2.19	5736	831
105ccT	90.0	2.0	3530	1263
99ccT	95.6	0	1647	440
95ccT	96.7	3.96	2740	892
94ccT	99.69	0	3899	1390
93ccT	93.76	2.45	3406	795
89ccT	99.69	0	4443	1839
85ccT	86.11	2.36	3286	859
79ccT	96.92	0.29	3629	868
67ccT	100.0	1.75	2453	816
57ccT	97.27	0.11	2121	627
53ccT	97.62	6.11	2615	619
52ccT	93.93	2.53	4062	1194

32ccT	98.37	1.63	2381	680
30ccT	67.56	8.28	3376	956
29ccT	94.87	0	2473	732
27ccT	96.77	0.97	3388	1026
22ccT	94.69	3.37	2999	807
19ccT	77.65	0.93	2502	781
6ccT	97.46	0.48	2148	477
5ccT	93.18	4.72	3028	702
2ccT	91.82	1.14	3655	879
113mtB	94.55	4.63	4294	1929
112mtB	94.91	0	2736	873
105mtB	90.54	1.42	2815	794
96mtB	99.4	1.25	2506	764
89mtB	98.71	0.81	3006	987
85mtB	99.28	0.24	4617	1341
61mtB	53.26	10.43	2695	852
59mtB	95.48	1.29	3343	1098
55mtB	95.86	2.53	3056	640
45mtB	77.72	8.28	3798	1093
43mtB	99.39	0	1772	468

33mtB	89.62	1.64	2331	493
18mtB	97.3	2.06	3221	845
16mtB	81.82	2.54	2159	799
7mtB	97.3	2.03	3209	823
4mtB	87.5	3.52	2747	948
4mxB	99.52	0.0	4270	1371
5mxB	96.76	4.55	3356	750
9mxB	92.44	3.36	3279	1128
10mxB	99.15	5.01	2735	1804
12mxB	93.96	1.81	3208	774
15mxB	93.83	1.71	2377	716
17mxB	90.0	0.18	3541	1333
19mxB	99.52	2.38	3301	889
21mxB	89.47	8.77	1025	337
26mxB	96.3	4.63	4227	1020
27mxB	99.13	1.0	3571	1213
29mxB	91.54	4.2	3316	1024
30mxB	97.37	8.77	3651	881
31mxB	98.76	0.0	3679	1055
32mxB	96.36	7.95	3623	1156

	34mxB	94.91	0.93	2872	886
	35mxB	98.86	3.41	4583	1010
	39mxB	99.38	2.88	4007	988
	46mxB	94.21	1.85	3485	1014
	47mxB	94.55	0.91	2433	854
	51mxB	97.8	0.0	3107	1099
	52mxB	98.42	3.32	2778	634
	56mxB	98.65	4.22	3845	1096
	61mxB	100	8.62	3150	961
	63mxB	93.02	2.29	3122	728
	81mxB	89.66	3.45	4156	1070
	87mxB	55.45	7.55	1653	261
	92mxB	77.91	3.51	3058	660
	99mxB	81.54	1.38	3822	1012
	101mxB	84.15	2.51	3291	820
	106mxB	71.02	2.5	2643	748
	116mxB	70.0	5.44	2727	592
	123mxB	54.84	3.39	2480	528
M6	99ccT	94.44	2.42	2473	1607

98ccT	60.89	0.96	3728	917
92ccT	68.52	3.01	2606	538
83ccT	99.06	0.94	4331	1155
81ccT	62.22	1.26	1259	331
79ccT	51.53	1.1	2098	657
75ccT	92.44	3.36	3220	1129
74ccT	92.13	1.57	2801	891
71ccT	93.04	3.86	3285	911
69ccT	93.78	2.15	3124	1811
66ccT	98.07	1.69	4759	1372
64ccT	98.57	0.48	2213	483
60ccT	81.88	0	2796	1201
53ccT	89.76	0	3978	1023
52ccT	96.19	3.39	2364	1423
51ccT	84.79	2.78	2097	473
48ccT	97.88	5.21	5013	1599
44ccT	72.17	3.12	3045	630
43ccT	98.31	4.66	2652	1844
36ccT	97.86	3.42	4008	1207
35ccT	98.81	1.79	2553	411

33ccT	100	1.65	3807	987
31ccT	93.45	1.71	2498	719
26ccT	98.54	1.81	3809	887
23ccT	86.45	1.89	5072	1229
20ccT	96.45	0.55	1963	609
15ccT	85.38	5.22	2463	824
12ccT	100	0.37	2458	401
10ccT	95.94	3.63	2951	1943
7ccT	99.69	0	3860	1399
6ccT	95.23	1.14	3730	887
5ccT	79.07	2.76	3043	609
3ccT	96.7	0.99	2683	893
2ccT	96.3	1.03	4131	1017
1ccT	98.1	5.87	2561	557
57mtB	100	0.94	3963	1447
53mtB	95.3	1.71	2433	915
52mtB	95.5	0	1754	465
40mtB	73.41	0	516	212
35mtB	94.96	2.32	3588	942
34mtB	98.37	2.44	1789	479

	32mtB	68.24	7.3	3791	1741
	28mtB	61.16	0.99	1040	323
	23mtB	94.91	0	2768	872
	20mtB	95.59	2.94	1421	263
	3mtB	90.91	2	3321	1240
	1mxB	99.01	0.49	2509	713
	33mxB	78.97	7.65	2520	1553
M8	173ccT	98.25	6.18	2583	726
	167ccT	87.24	0.07	2901	965
	157ccT	98.88	1.12	2670	932
	150ccT	96	2.36	2175	609
	149ccT	95.6	0.55	1631	434
	144ccT	84.77	9.51	3969	514
	140ccT	97.27	5.18	3476	1196
	136ccT	100	5.36	5265	1928
	134ccT	95.62	0.2	2029	589
	131ccT	92.69	3.35	1768	436
	130ccT	94.77	1.3	2514	677
	125ccT	80.88	5.8	1283	168
	114ccT	96.1	0.16	2746	715

113ccT	90.16	0.04	3807	1203
106ccT	99.15	2.56	2373	650
105ccT	97.66	0.73	2913	767
104ccT	76.19	9.5	2812	1073
92ccT	68.32	9.87	1819	231
91ccT	93.1	2.77	3571	963
90ccT	90.11	4.27	2423	332
87ccT	98.11	0.07	2665	498
81ccT	98.57	3.1	2981	573
77ccT	96.99	0.55	2377	648
71ccT	52.97	0	2106	460
70ccT	80.62	0	3048	677
69ccT	99.62	1.15	2147	561
67ccT	63.64	4.19	3471	1039
56ccT	97.61	0.47	2364	735
38ccT	94.68	0.81	4006	963
35ccT	96.36	6.09	4585	1651
34ccT	60.67	2.7	3019	595
30ccT	96.45	0.55	1942	607
23ccT	59.7	2.59	2494	560

18ccT	99.84	2.1	4153	1219
13ccT	72.28	2.56	3486	994
9ccT	74.23	3.59	1921	416
8ccT	89.34	5.58	3116	747
3ccT	98.65	3.03	3335	829
92mtB	92.74	1.71	2327	716
81mtB	56.07	0.61	694	187
64mtB	95.42	2.5	2229	646
60mtB	83.65	2.67	2044	626
52mtB	97.7	0	2206	774
48mtB	90	2	3122	1052
45mtB	88.43	1.2	4881	1094
29mtB	98.4	0.77	1800	250
26mtB	79.09	4.55	2576	914
25mtB	94.15	1.11	2467	731
21mtB	62.98	8.64	2850	1044
18mtB	97.73	0.2	2933	600
14mtB	99.19	1.22	1764	480
12mtB	98.39	0.97	5106	1018
3mtB	94.68	3.11	2395	1500

	1mxB	92.73	2.91	3782	1191
	2mxB	94.44	2.14	2944	1832
	4mxB	99.15	2.47	2583	1672
	6mxB	100	1.75	2075	791
	10mxB	70.53	4.31	889	261
	11mxB	98.17	2.66	3066	681
	15mxB	98.37	3.86	2675	734
	17mxB	99.16	0.56	3165	958
	21mxB	94.44	2.78	3291	747
	22mxB	92.01	7.55	3282	757
	28mxB	99.52	1.76	2628	688
	30mxB	96.63	1.12	1938	536
	39mxB	100	0.5	3455	1920
	57mxB	96.68	5.98	4041	1170
M9	157ccT	94.91	0.93	2528	726
	150ccT	93.29	0.99	2985	705
	145ccT	59.07	1.4	3674	1051
	141ccT	98.29	0	2857	638
	140ccT	89.08	3.36	3200	1123
	138ccT	99.52	0	4268	1375

137ccT	59.93	1.22	2122	489
135ccT	74.84	0.18	4238	1237
123ccT	98.72	4.7	3350	1977
119ccT	92.63	3.7	2321	544
118ccT	95.23	1.14	4142	930
113ccT	91.82	9.27	4084	1076
112ccT	90.92	7.92	4102	1220
107ccT	96.58	1.71	2656	925
106ccT	78.58	0	1550	580
105ccT	95.34	6.36	2814	1782
101ccT	75.07	2.97	2890	858
100ccT	63.86	8.61	1308	454
95ccT	96.64	0.42	2570	745
94ccT	98.68	3.3	5478	1589
90ccT	51.63	3.85	2439	878
89ccT	85.36	2.76	4350	903
88ccT	98.68	1.98	3732	1479
87ccT	94.07	4.2	3361	1031
86ccT	90.48	2.72	2690	658
79ccT	97.03	7.2	2779	1915

76ccT	91.78	1.99	6004	1703
75ccT	95.37	0.93	2954	933
73ccT	98.71	0.81	3124	1000
67ccT	96.77	1.29	3342	1098
66ccT	94.66	2.14	4184	2039
62ccT	95.91	2.3	3963	869
54ccT	97.27	4.73	3436	1184
53ccT	96.15	7.26	2521	777
40ccT	98.77	0.49	2503	735
38ccT	96.45	0.55	1931	607
36ccT	92.27	0.91	3884	1366
31ccT	99.19	0.81	1871	468
30ccT	63.58	2.44	2462	400
26ccT	86.78	1.15	3956	997
20ccT	100	0	1416	524
18ccT	88.17	0.65	2405	693
17ccT	99.32	4.08	3317	881
7ccT	96.55	1.15	3461	789
6ccT	93.79	3.42	2136	684
3ccT	96.7	4.4	2366	712

2ccT	92.73	2.91	3084	906
1ccT	92.73	7	3024	1070
53mtB	81.46	4.51	2605	659
51mtB	99.37	6.18	2580	725
29mtB	98.65	2.7	3246	842
2mxB	94.91	0.0	2857	871
3mxB	100	1.75	2002	790
8mxB	99.69	0.94	4428	1822
10mxB	97.97	2.03	3291	829
11mxB	93.13	2.78	3473	1105
14mxB	97.8	0.0	3068	1102
15mxB	94.87	0.85	2579	734
19mxB	99.13	0.9	3579	1213
23mxB	98.06	0.0	1702	363
25mxB	91.43	2.47	3103	944
27mxB	75.91	2.27	1926	808
35mxB	96.7	1.21	2740	916
36mxB	94.78	5.38	3319	998
37mxB	99.53	0.94	6001	1377
41mxB	99.09	4.55	4215	1927

	43mxB	100	0.91	1991	593
	45mxB	97.09	0.97	2180	420
	47mxB	90.74	1.85	3119	801
	48mxB	95.25	3.1	2165	537
	52mxB	91.2	3.4	2970	982
	85mxB	64.38	0.94	4299	1201
	86mxB	71.93	6.83	4434	969
	93mxB	62.27	8.15	6876	947
M11	186ccT	98.43	3.42	4635	1209
	185ccT	86.36	1.72	3503	754
	183ccT	87.86	0.97	1920	412
	176ccT	89.24	5.21	5038	1561
	166ccT	99.37	5.62	2588	728
	159ccT	94.28	6.21	1991	1321
	158ccT	92.44	3.36	3241	1138
	155ccT	81.77	3.35	2553	528
	154ccT	93.17	0.55	1841	524
	153ccT	97.81	2.03	4191	1202
	152ccT	99.69	1.65	3881	990
	149ccT	93.94	0.91	2339	1206

138ccT	66.05	5.16	2988	753
134ccT	77.66	5.17	4168	1014
126ccT	95.3	3.42	2357	720
125ccT	94.58	2.07	2127	776
105ccT	71.66	3.03	2061	559
102ccT	95.95	3.72	3282	780
98ccT	94.17	0.99	2630	853
97ccT	78.04	0	763	89
89ccT	99.44	0	2502	843
85ccT	76.49	6.13	3295	573
78ccT	98.65	3.25	2763	700
75ccT	98.5	0.43	3257	1995
69ccT	99.69	0	3856	1382
68ccT	93.27	0	2827	1236
64ccT	97.97	1.63	2314	723
56ccT	96.86	3.15	2759	670
52ccT	95.73	1.71	2515	857
47ccT	54.38	1.15	3781	700
39ccT	53.75	0.07	2381	499
35ccT	83	5.5	3376	1236

31ccT	93.79	3.42	2129	685
29ccT	68.81	0.99	1257	265
24ccT	100	8.62	3961	1049
23ccT	76.87	0	4338	710
18ccT	72.67	0	2542	456
17ccT	90.65	0	3425	909
13ccT	81.6	3.02	3439	923
12ccT	66.26	4.16	3236	996
9ccT	87.1	2.53	1833	399
8ccT	93.98	0.65	2626	878
123mtB	96.2	2.73	3309	817
116mtB	97.79	0.74	2667	506
115mtB	98.06	0	1597	344
112mtB	98.39	0	1426	351
110mtB	97.27	5.18	3475	1204
109mtB	97.8	4.4	2398	711
104mtB	84.6	3.32	2190	529
101mtB	96.21	0	3121	970
100mtB	98.03	0.1	2910	623
94mtB	97.3	2.73	3247	846

92mtB	95.7	1.1	3147	853
91mtB	98.81	1.4	2446	738
85mtB	90.29	0.97	2414	368
76mtB	62.91	0	688	184
69mtB	82.83	7.93	3767	1301
65mtB	86.41	9.71	3329	373
61mtB	96.45	1.64	1964	621
52mtB	94.91	0	2760	873
44mtB	87.77	0	1639	464
43mtB	96.58	1.71	2434	933
39mtB	71.27	4.41	1764	363
29mtB	95.49	1.5	3585	2048
27mtB	100	0.91	1990	597
26mtB	95.89	0.63	1614	335
24mtB	57.56	9.77	2539	841
22mtB	90.91	2.91	3301	1241
21mtB	64.85	0.2	1115	336
19mtB	78.65	1.33	2074	630
18mtB	89.55	4.27	2570	1020
9mtB	96.62	0.62	3276	816

	1mxB	95.73	0.41	1724	471
	3mxB	94.07	5.44	2557	1735
	6mxB	96.61	8.19	2749	1750
	12mxB	99.52	0.56	4453	1375
	14mxB	99.51	0.99	2682	679
	16mxB	92.73	1.09	3000	915
	17mxB	98.72	5.32	3730	1180
	20mxB	99.51	0.49	3186	759
	23mxB	98.13	1.87	2818	1283
	24mxB	97.47	1.68	3483	872
	25mxB	93.73	1.54	2417	887
	30mxB	97.3	3.38	2862	855
	39mxB	99.01	4.31	2594	774
	40mxB	94.87	0.0	2532	733
	46mxB	99.09	0.91	3203	972
	52mxB	99.09	1.09	3228	987
	56mxB	97.97	1.1	1599	371
	86mxB	91.11	5.19	4828	1165
M13	172ccT	99.13	0.91	3571	1222
	168ccT	97.51	2.05	3785	1009

160ccT	99.52	0.24	4296	1375
158ccT	93.53	1.72	2740	813
157ccT	91.78	3.36	3262	1132
151ccT	89.32	1.97	3031	666
148ccT	99.09	3.64	4103	1910
144ccT	88.82	2.64	3356	805
143ccT	99.38	1.85	3866	976
141ccT	96.96	0.91	2700	518
135ccT	65.95	3.83	4853	1134
132ccT	98.72	5.98	3923	1207
116ccT	92.05	2.84	3813	1166
113ccT	75.45	2.91	3628	908
111ccT	74.04	2.08	3729	1532
108ccT	51.21	1.87	2887	662
106ccT	86.16	4.96	3284	1762
99ccT	75.71	4.49	1162	251
97ccT	95.7	4.84	5046	1567
96ccT	75.71	9.33	3727	1007
92ccT	98.65	4.05	3713	987
84ccT	72.71	2.86	2795	831

83ccT	75.86	3.47	2378	701
81ccT	88.56	6.98	5925	1795
79ccT	98.54	2.29	3489	911
72ccT	92.73	0.18	3525	1363
70ccT	96.7	2.75	3573	1140
69ccT	89.06	7.47	2560	491
65ccT	74.83	0.38	2281	423
63ccT	80.52	3.77	3191	1014
61ccT	90.61	2	3544	1257
57ccT	86.88	4.78	3865	770
50ccT	99.53	2.36	5076	1488
48ccT	96.29	0.75	3685	1110
47ccT	92.64	0.91	5554	1668
45ccT	92.21	3.23	5524	965
42ccT	96.48	3.26	2908	874
41ccT	95.73	1.71	2538	919
40ccT	69.92	9.12	3080	698
34ccT	96.76	4.55	3878	778
32ccT	53.94	0.95	842	112
25ccT	95.4	0	3705	1079

21ccT	96.59	1.14	2704	626
20ccT	97.59	2.08	2622	602
12ccT	82.48	1.71	3023	547
10ccT	96.45	1.64	1963	608
3ccT	90.09	6.09	2939	1912
96mtB	96.86	0	4295	1802
94mtB	62.79	6.04	5557	1231
88mtB	92.13	0	2679	804
84mtB	62.17	3.64	2681	820
79mtB	98.18	0.91	3915	1386
68mtB	97.27	0.91	5312	1794
65mtB	96.24	5.38	3315	985
41mtB	95.23	1.14	3519	876
35mtB	94.51	3.91	2322	643
26mtB	69.72	2.56	2437	834
17mtB	95.53	1.36	1741	485
15mtB	96.86	1.89	3876	1431
11mtB	87.27	6.09	3076	1141
7mtB	92.47	0	3157	673
1mxB	98.28	8.62	2754	716

7mxB	94.28	2.19	3699	1056
8mxB	96.23	7.68	3479	707
10mxB	95.96	3.64	3403	826
11mxB	99.4	1.25	2597	764
13mxB	96.21	4.44	3124	1078

Table S3. BLASTp and KOfamKO functional annotations of selected hydrolase candidates with high predicted melting temperatures.

MAG ID	Closest GTDB taxon	Sequence ID	Best BLASTp hit (% identity)	KO ID	KOfamKO E-value	KO Definition	Protein length (aa)	Predicted Tm (°C)
M5120ccT	*Meiothermus*	k141_82157_33	alpha/beta fold hydrolase (99.30%)	K10216*	1.2e-106	2-hydroxymuconate-semialdehyde hydrolase [EC:3.7.1.9]	265	82.7
		k141_180943_7	alpha-amylase family glycosyl hydrolase (96.46%)	K01208	7.4e-153	cyclomaltodextrinase / maltogenic alpha-amylase / neopullulanase [EC:3.2.1.54	514	82.1

						3.2.1.133 3.2.1.135]		
		k141_253107_89	glycoside hydrolase family 13 protein (97.75)	K01208	8.8e-137	cyclomaltodextrinase / maltogenic alpha-amylase / neopullulanase [EC:3.2.1.54 3.2.1.133 3.2.1.135]	563	77.9
		k141_856_1	glycoside hydrolase family 125 protein (91.99%)	K09704	2.1e-33	uncharacterized protein	558	77.2
		k141_22697_128	TldD/PmbA family protein (97.20%)	K03568*	2e-147	TldD protein	464	76.6
M643ccT	*Thermus antranikianii*	k141_129750_23	nitrilase-related carbon-nitrogen hydrolase (97.22%)	K12251	5.5e-50	N-carbamoylputrescine amidase [EC:3.5.1.53]	283	87.6
		k141_84105_4	MBL fold metallo-hydrolase (98.25%)	K13075	1.2e-29	N-acyl homoserine lactone hydrolase [EC:3.1.1.81]	285	85.1
		k141_123823_67	alpha/beta hydrolase (98.08%)	K07214	8.2e-33	iron(III)-enterobactin esterase [EC:3.1.1.108]	312	85.0
		k141_44311_31	carbon-nitrogen hydrolase	K12251	8.1e-17	N-carbamoylputrescine	282	84.9

			family protein (97.87%)			amidase [EC:3.5.1.53]		
		k141_44311_71	P1 family peptidase (93.52%)	K18572	6.5e-82	putative pantetheine hydrolase [EC:3.5.1.-]	476	84.5
M839mxB	*Calidithermus chliarophilus*	k141_434866_18	glucan 1,4-alpha-glucosidase (94.56%)	K01178 *	1.9e-91	glucoamylase [EC:3.2.1.3]	762	82.4
		k141_417447_45	alpha-amylase family glycosyl hydrolase, partial (97.53)	K01208	1.4e-153	cyclomaltodextrinase / maltogenic alpha-amylase / neopullulanase [EC:3.2.1.54 3.2.1.133 3.2.1.135]	510	81.0
		k141_92092_3	glycoside hydrolase family 125 protein (92.89%)	K09704	6.3e-32	uncharacterized protein	583	79.3
		k141_291505_43	NfeD family protein (95.07%)	K07403 *	1.8e-112	membrane-bound serine protease (ClpP class)	420	78.6
		k141_175578_5	glycoside hydrolase family 31 protein (97.32%)	K05546	1.9e-173	mannosyl-oligosaccharide alpha-1,3-glucosidase [EC:3.2.1.207]	788	78.4
M966ccT	*Meiothermus*	k141_148009_20	glucan 1,4-alpha-glucosidase (93.39%)	-	-	-	255	79.5

		k141_111401_20	beta-ureidopropionase (99.06%)	K12251	8e-49	N-carbamoylputrescine amidase [EC:3.5.1.53]	287	78.0
		k141_195287_5	alpha-amylase (99.62%)	K01187*	2.7e-128	alpha-glucosidase [EC:3.2.1.20]	531	71.5
		k141_24680_7	endopeptidase La (93.17%)	K01338*	0	ATP-dependent Lon protease [EC:3.4.21.53]	792	69.8
M11 29mtB	*Meiothermus_B silvanus*	k141_71355_57	nitrilase-related carbon-nitrogen hydrolase (97.22%)	K12251	5.5e-50	N-carbamoylputrescine amidase [EC:3.5.1.53]	283	87.6
		k141_489978_7	glycoside hydrolase family 15 protein (93.43%)	K01178*	1.2E-89	glucoamylase [EC:3.2.1.3]	763	80.4
		k141_482068_13	glycoside hydrolase family 13 protein (98.57%)	K01208	2.5e-157	cyclomaltodextrinase / maltogenic alpha-amylase / neopullulanase [EC:3.2.1.54 3.2.1.133 3.2.1.135]	516	78.3
M13 148ccT	*Caldilinea*	k141_251414_593	acetyl-CoA hydrolase/transferase family protein (97.67%)	K18122	3.5e-197	4-hydroxybutyrate CoA-transferase [EC:2.8.3.-]	418	62.5
		k141_241632_91	SDH family Clp fold serine	K07403	6e-20	membrane-bound serine	267	61.4

			proteinase (100%)			protease (ClpP class)		
M652mtB	*Sulfolobales*	k141_203484_11	adenosylhomocysteinase (79.14%)	K01251*	8.9e-171	adenosylhomocysteinase [EC:3.13.2.1]	413	79.7
M119ccT	*Sulfolobales*	k141_325407_2	hypothetical protein ATG_14710 (79.95%)	K01251*	2.8e-171	adenosylhomocysteinase [EC:3.13.2.1]	413	79.4

Hydrolase candidates correspond to selected sequences from the MAGs presented in Table 8 and were prioritized according to their predicted melting temperature (Tm). Best BLASTp hits indicate the closest functional annotation obtained against the NCBI ClusteredNR database, whereas KOfamKO annotations provide complementary KO-based functional assignments. Asterisks (*) indicate hits above the KOfamKOALA reporting threshold. In cases where BLASTp and KOfamKO annotations differed, both annotations are reported to illustrate uncertainty associated with sequence-based functional prediction.

Table S4. Source organisms represented in the custom thermophile-derived hydrolase reference database. The table summarizes the number and relative proportion of protein records assigned to each source genus represented in the database.

Source organism	Records
Thermus thermophilus	312
Thermus scotoductus	265
Geobacillus stearothermophilus	161
Streptococcus thermophilus	136
Parageobacillus thermoglucosidasius	100
Geobacillus thermoleovorans	74
Thermus hydrothermalis	70
Thermus composti	67
Geobacillus thermodenitrificans	66
Thermus arciformis	61
Thermus thermamylovorans	55
Thermococcus thioreducens	54
Thermus thalpophilus	50

Thermus sp. LT1-2-5	48
Thermus filiformis	45
Thermus brockianus	45
Thermobacillus sp.	45
Thermus oshimai	43
Thermotoga maritima MSB8	40
Bifidobacterium thermophilum	38
Thermococcus barophilus	36
Saccharopolyspora thermophila	36
Chloracidobacterium thermophilum	35
Gandjariella thermophila	35
Thermus parvatiensis	34
Thermus caliditerrae	33
Thermococcus sp.	31
Thermococcus siculi	29
Geobacillus subterraneus	28
Vulcaniibacterium thermophilum	26
Thermus sp. PS18	25
Geobacillus zalihae	25
Thermococcus chitonophagus	24

Thermotoga maritima	23
Thermobacillus xylanilyticus	22
Thermobacillus composti KWC4	20
Sphingobacterium thermophilum	20
Pyrococcus furiosus DSM 3638	20
Thermus tengchongensis	19
Thermus aquaticus	19
Geobacillus thermocatenulatus	19
Thermococcus peptonophilus	18
Azospirillum thermophilum	18
Thermus antranikianii	17
Thermococcus profundus	17
Thermococcus litoralis DSM 5473	17
Thermococcus kodakarensis KOD1	17
Dictyoglomus thermophilum	17
Schleiferia thermophila	17
Thermococcus argininiproducens	16
Thermococcus litoralis	16
Thermotoga sp. SG1	16
Thermotoga sp. RQ7	16

Thermothelomyces thermophilus ATCC 42464	16
Thermococcus nautili	15
Thermoflavifilum thermophilum	15
Thermococcus sibiricus MM 739	14
Thermococcus onnurineus NA1	14
Geobacillus stearothermophilus ATCC 12980	13
Thermococcus kodakarensis	12
Geobacillus stearothermophilus 10	12
Thermotoga sp. 38H-to	12
Hyperthermus butylicus	11
Thermobacillus composti	11
Thermococcus onnurineus	11
Thermococcus gammatolerans EJ3	11
Schleiferia thermophila str. Yellowstone	11
Ureibacillus thermophilus	10
Hyperthermus butylicus DSM 5456	9
Geobacillus thermoleovorans CCB_US3_UF5	9
Pseudonocardia thermophila	9
Streptococcus thermophilus MTH17CL396	9
Thermococcus radiotolerans	9

Thermotoga neapolitana DSM 4359	9
Geobacillus thermopakistaniensis	9
Geobacillus proteiniphilus	9
Pyrococcus kukulkanii	9
Pyrococcus yayanosii CH1	9
Thermotoga sp. Ku-13t	9
Thermococcus barophilus MP	8
Thermococcus sibiricus	8
Flavobacterium thermophilum	8
Methanosarcina thermophila	8
Streptococcus thermophilus M17PTZA496	8
Streptococcus thermophilus TH1477	8
Thermotoga sp. KOL6	8
Thermobacillus sp. ZCTH02-B1	8
Parageobacillus yumthangensis	8
Streptococcus thermophilus TH982	7
Methanopyrus kandleri AV19	7
Thermococcus camini	7
Thermococcus sp. 5-4	7
Geobacillus kaustophilus GBlys	7

Geobacillus genomosp. 3	7
Parageobacillus galactosidasius	7
Thermotoga sp. RQ2	7
Bifidobacterium thermophilum RBL67	6
Dictyoglomus thermophilum H-6-12	6
Streptococcus thermophilus MTCC 5460	6
Streptococcus thermophilus MTCC 5461	6
Thermococcus aciditolerans	6
Geobacillus kaustophilus	6
Thermococcus indicus	6
Parageobacillus thermantarcticus	6
Hydrogenovibrio thermophilus	6
Parageobacillus toebii	6
Thermus aquaticus Y51MC23	5
Thermus oshimai JL-2	5
Geobacillus thermodenitrificans NG80-2	5
Dissulfurispira thermophila	5
Pseudozobellia thermophila	5
Natrinema sp. CBA1119	5
Novibacillus thermophilus	5

Streptococcus thermophilus ASCC 1275	5
Streptococcus thermophilus TH985	5
Paenibacillus macerans	5
Methanopyrus kandleri	5
Pyrolobus fumarii 1A	5
Thermotoga sp. Mc24	5
Thermotoga sp. TBGT1766	5
Symbiobacterium thermophilum	5
Thermococcus eurythermalis	5
Parageobacillus sp. KH3-4	5
Coralloluteibacterium thermophilum	5
Thermus thermophilus HB8	4
Geobacillus thermoleovorans B23	4
Methanosarcina thermophila MST-A1	4
Streptococcus thermophilus 1F8CT	4
Pyrococcus furiosus COM1	4
Thermotoga sp. 2812B	4
Thermotoga sp. EMP	4
Thermus thermophilus HB27	4
Streptococcus thermophilus MN-ZLW-002	4

Thermotoga sp. Cell2	4
Geobacillus kaustophilus HTA426	4
Geobacillus kaustophilus NBRC 102445	4
Thermococcus pacificus	4
Thermococcus barossii	4
Thermococcus gorgonarius	4
Vulcanisaeta thermophila	4
Sporolituus thermophilus DSM 23256	4
Thermotoga sp. Xyl54	4
Thermotoga petrophila RKU-10	4
Thermococcus gammatolerans	3
Methanonatronarchaeum thermophilum	3
Fontimonas thermophila	3
Saccharococcus thermophilus	3
Hydrogenobacter thermophilus	3
Pyrococcus furiosus	3
Thermus thermophilus SG0.5JP17-16	3
Thermus scotoductus SA-01	3
Symbiobacterium thermophilum IAM 14863	3
Thermotoga neapolitana LA10	3

Geobacillus sp. GHH01	3
Thermococcus sp. P6	3
Parageobacillus sp. G301	3
Methanosarcina thermophila TM-1	3
Streptococcus thermophilus TH1435	3
Natranaerobius thermophilus JW/NM-WN-LF	3
Hydrogenobacter thermophilus TK-6	3
Thermotoga petrophila RKU-1	3
Thermus sp.	3
Geobacillus sp. PA-3	3
Sphaerobacter thermophilus	3
Thermococcus thermotolerans	2
Thermococcus sp. 18S1	2
Desulfurispora thermophila	2
Spirochaeta thermophila	2
Streptococcus thermophilus CAG:236	2
Streptococcus thermophilus CNRZ1066	2
Streptococcus thermophilus LMG 18311	2
Geobacillus stearothermophilus ATCC 7953	2
Rubellimicrobium thermophilum DSM 16684	2

Thermochaetoides thermophila	2
Thermococcus celer	2
Thermococcus sp. 21S7	2
Thermococcus sp. EP1	2
Thermococcus piezophilus	2
Parageobacillus thermoglucosidasius NBRC 107763	2
Parageobacillus thermoglucosidasius C56-YS93	2
Methanosarcina thermophila CHTI-55	2
Jhaorihella thermophila	2
Anaerolinea thermophila UNI-1	2
Streptococcus thermophilus TH1436	2
Tepidiphilus thermophilus	2
Dissulfuribacter thermophilus	2
Pyrococcus sp. NA2	2
Thermus caldilimi	2
Rubellimicrobium thermophilum	2
Thermococcus aggregans	2
Thermococcus zilligii	2
Penicillium oxalicum	2
Thermococcus sp. GR6	2

Thermococcus sp. M36	2
Thermococcus sp. LS1	2
Thermococcus sp. 21S9	2
Thermococcus sp. SY098	2
Geobacillus sp. 44B	2
Geobacillus sp. LEMMY01	2
Geobacillus sp. 46C-IIa	2
Parageobacillus	2
Crassaminicella thermophila	2
Geobacillus lituanicus	2
Stenotrophomonas thermophila	2
Bacillus thermoproteolyticus	2
Thermus sp. CCB_US3_UF1	1
Hyperthermus sp.	1
Tepidiforma thermophila	1
Qipengyuania thermophila	1
Xylanivirga thermophila	1
Deferribacter thermophilus	1
Sporolituus thermophilus	1
Methanopyrus sp. SNP6	1

Methanopyrus sp. KOL6	1
Pyrolobus fumarii	1
Thermotoga sp. TBYP3.1.4.1	1
Chloracidobacterium thermophilum B	1
Thermothelomyces thermophilus	1
Streptococcus thermophilus ND03	1
Geobacillus sp. JS12	1
Thermococcus guaymasensis DSM 11113	1
Geobacillus jurassicus	1
Parageobacillus toebii NBRC 107807	1
Parageobacillus caldoxylosilyticus NBRC 107762	1
Tetrahymena thermophila	1
Spirochaeta thermophila DSM 6578	1
Anaerolinea thermophila	1
Hydrogenimonas thermophila	1
Streptococcus thermophilus JIM 8232	1
Sphaerobacter thermophilus DSM 20745	1
Thermus brevis	1
Thermus igniterrae	1
Thermus albus	1

Thermus sp. NMX2.A1	1
Thermus sp. 2.9	1
Thermodesulfobacterium thermophilum	1
Caldimonas thermodepolymerans	1
Thermococcus waiotapuensis	1
Thermococcus paralvinellae	1
Thermococcus sp. CX2	1
Thermococcus alcaliphilus	1
Thermococcus stetteri	1
Thermococcus sp. ES12	1
Thermococcus sp. 9N3	1
Thermococcus sp. MV11	1
Thermococcus sp. MV5	1
Thermococcus sp. MAR1	1
Thermococcus sp. JdF3	1
Thermococcus sp. Bubb.Bath	1
unclassified Thermococcus	1
Thermococcus cleftensis	1
Thermococcus henrietii	1
Geobacillus sp. 47C-IIb	1

Geobacillus	1
Parageobacillus sp. VR-IP	1
Parageobacillus genomosp. 1	1
Spirochaeta thermophila DSM 6192	1
Pyrococcus horikoshii	1
Pyrococcus woesei	1
Pyrococcus yayanosii	1
Pyrococcus sp. ST04	1
Pyrococcus abyssi	1
Geobacillus sp. Y412MC52	1
Geobacillus sp. ZH1	1
Geobacillus sp. C56-T3	1
Geobacillus sp. B4113_201601	1
Geobacillus sp. PK12	1
Geobacillus sp. TFV-3	1
Parageobacillus sp. SY1	1
Lihuaxuella thermophila	1
Lutispora thermophila DSM 19022	1
Thermococcus celericrescens	1
Thermococcus sp. JCM 11816	1

Geobacillus sp. 44C	1
Thermotoga petrophila	1

The database included proteins from organisms containing thermophile-associated taxonomic designations or experimentally reported thermophilic representatives.

Table S5. Broad hydrolase categories represented in the custom thermophile-derived hydrolase reference database. The table summarizes the 3392 NCBI protein records according to normalized annotation descriptions grouped into broad hydrolase categories.

Broad enzyme category	Records
Other/generic hydrolase	1562
Protease/peptidase	557
Esterase/carboxylesterase	515
Polysaccharide-degrading / pectinase-related	399
Lipase/phospholipase	359

M5

gtdbtk.ar53.classify-trimmed.tree

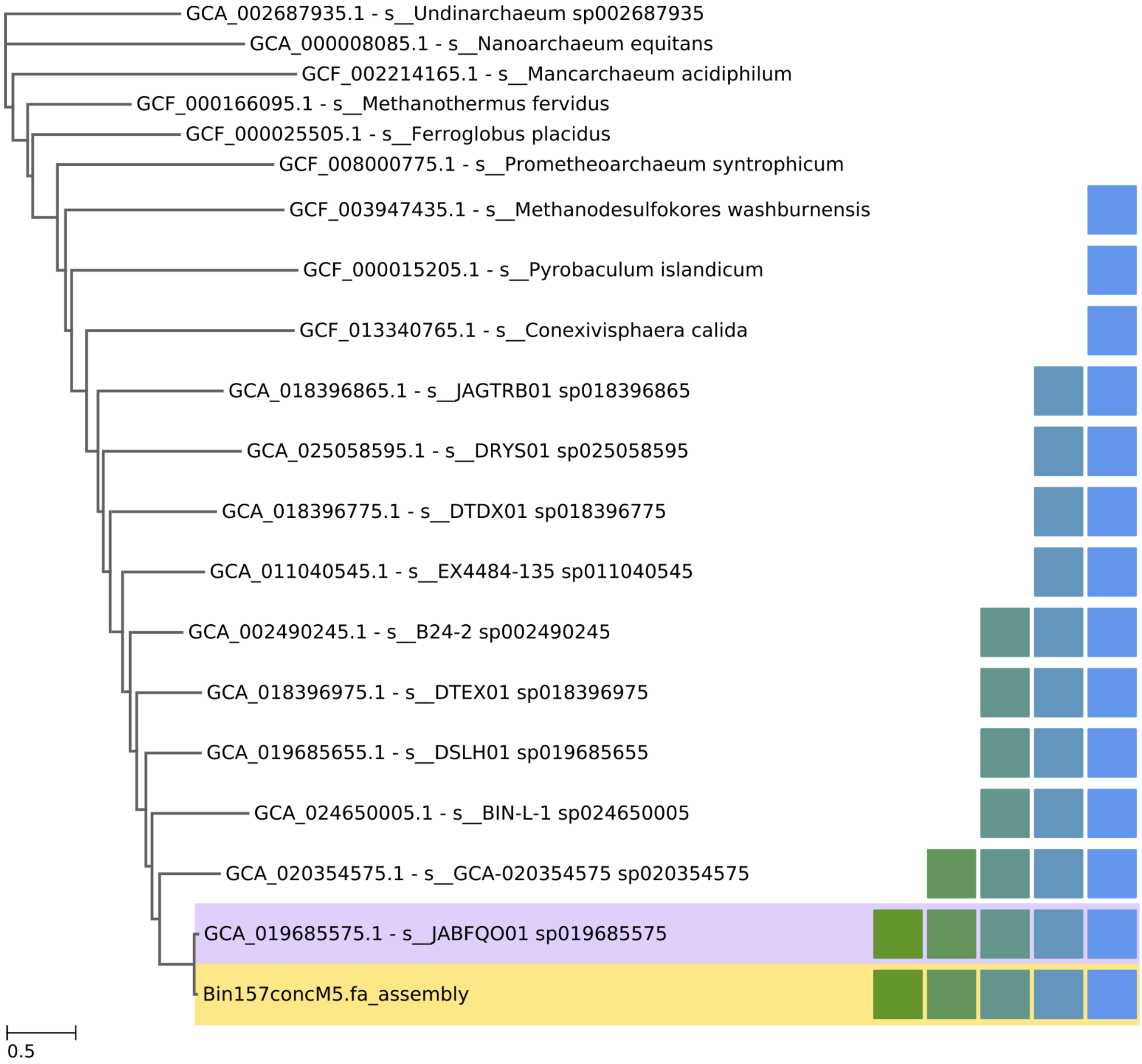

M6

gtdbtk.ar53.classify-trimmed.tree

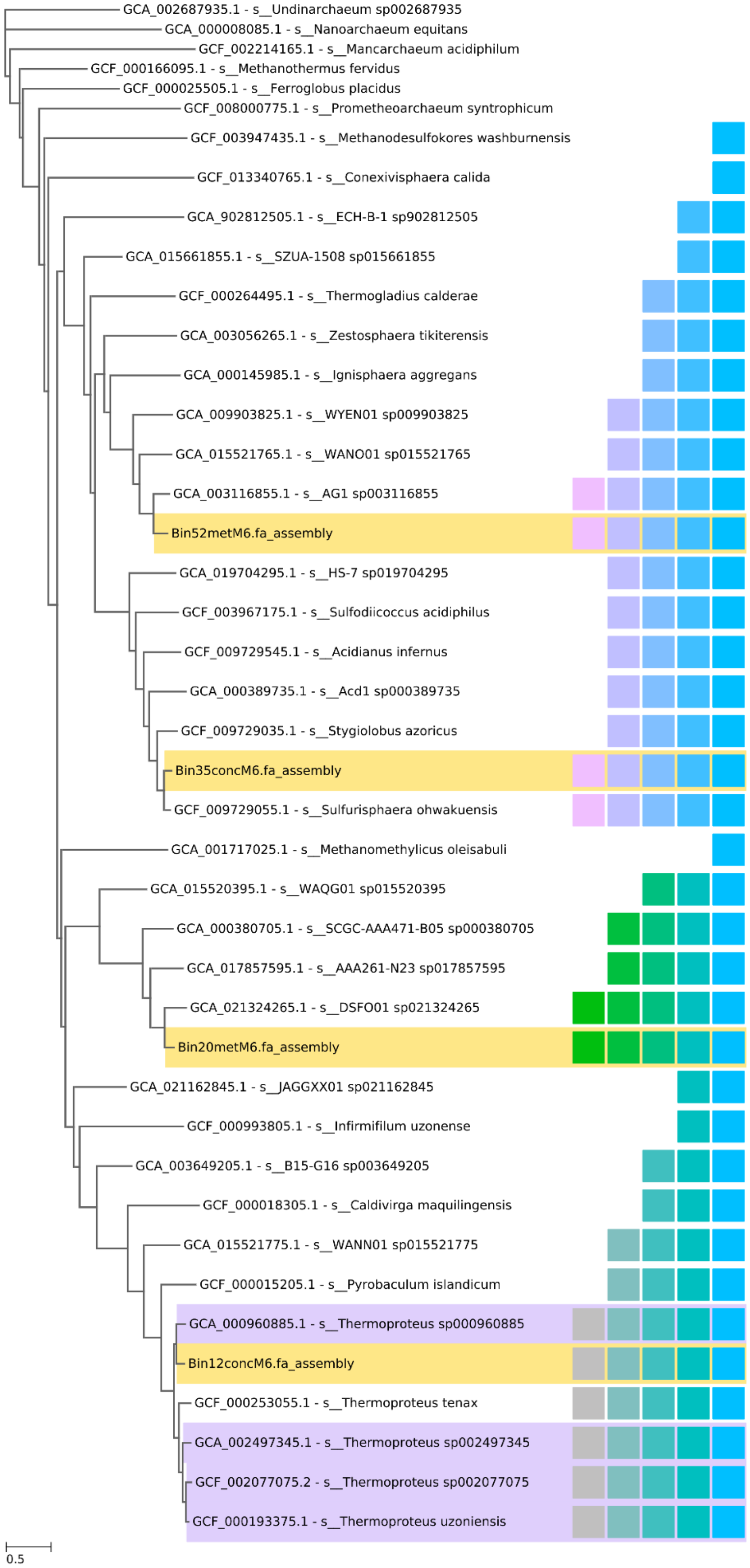

M8

gtdbtk.ar53.classify-trimmed.tree

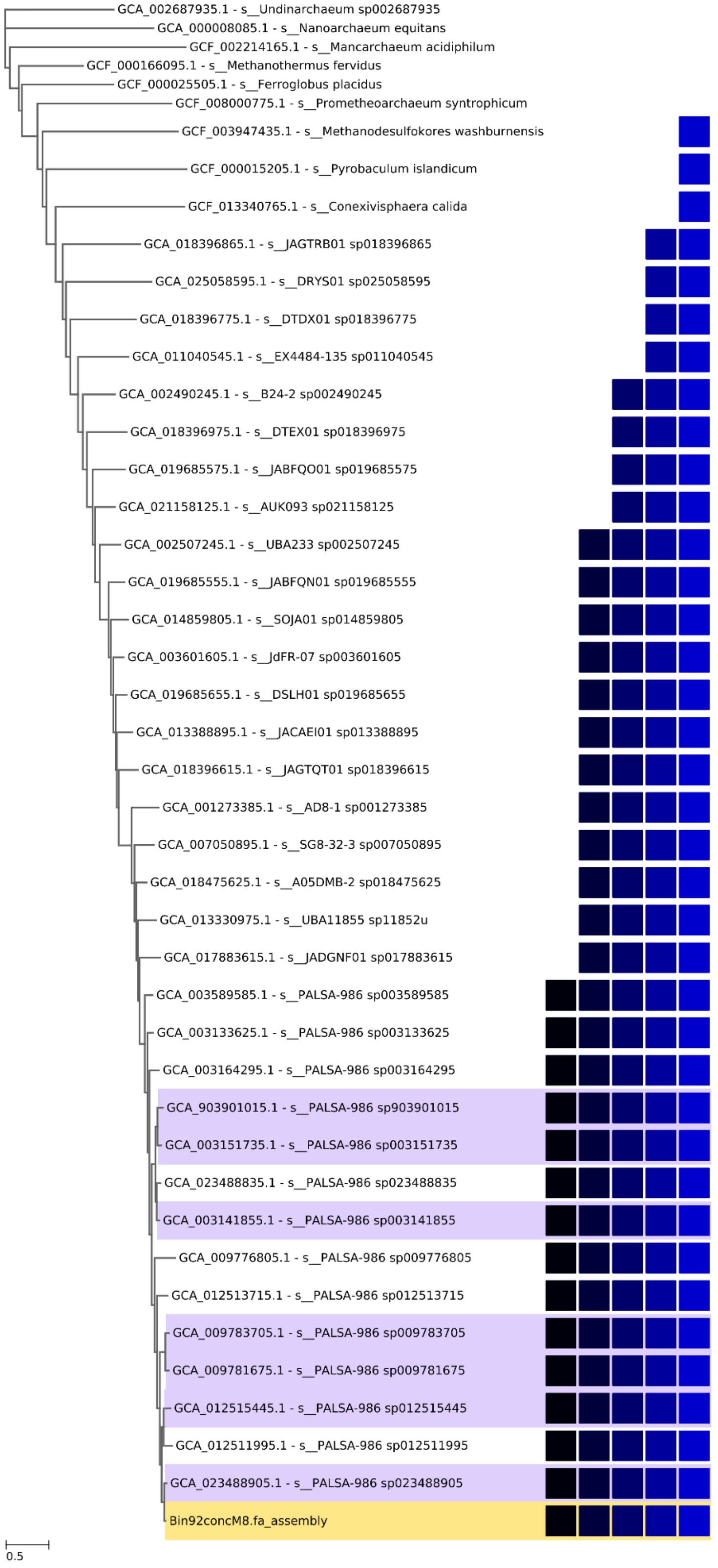

M9

gtdbtk.ar53.classify-trimmed.tree

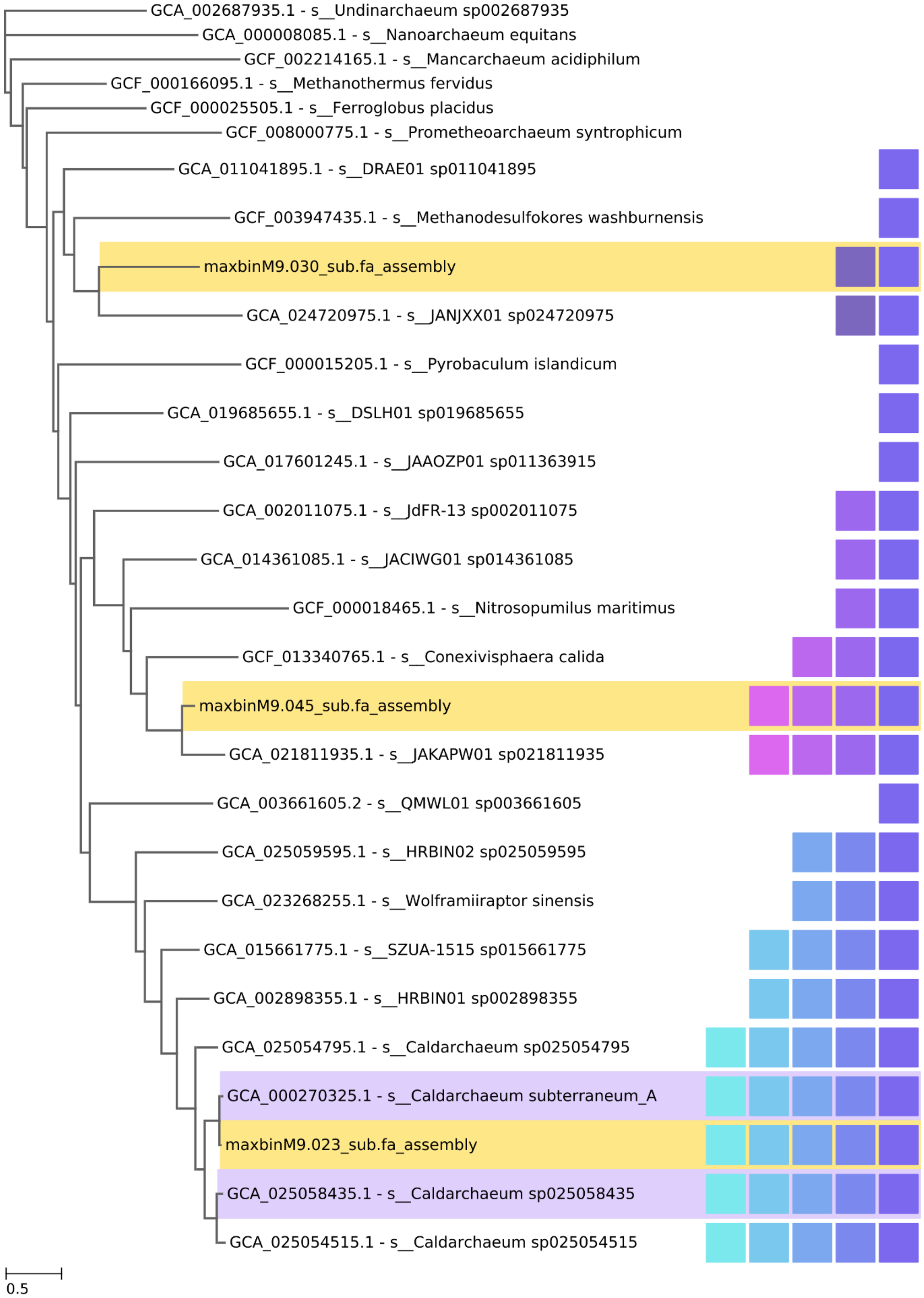

M11

gtdbtk.ar53.classify-trimmed.tree

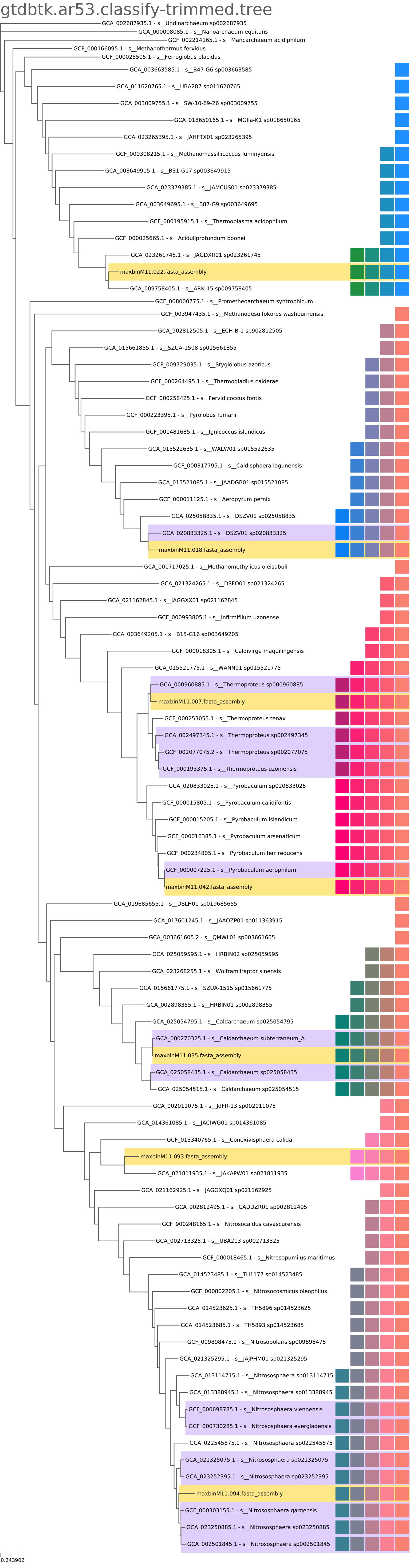

gtdbtk.backbone.bac120.classify-trimmed.tree

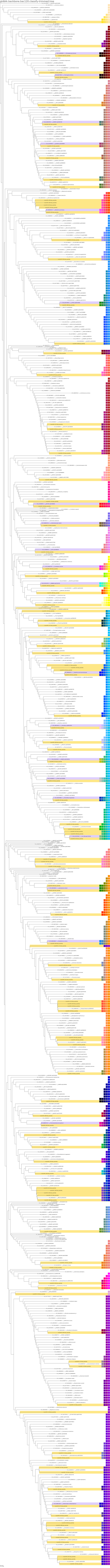

M13

gtdbtk.ar53.classify-trimmed.tree

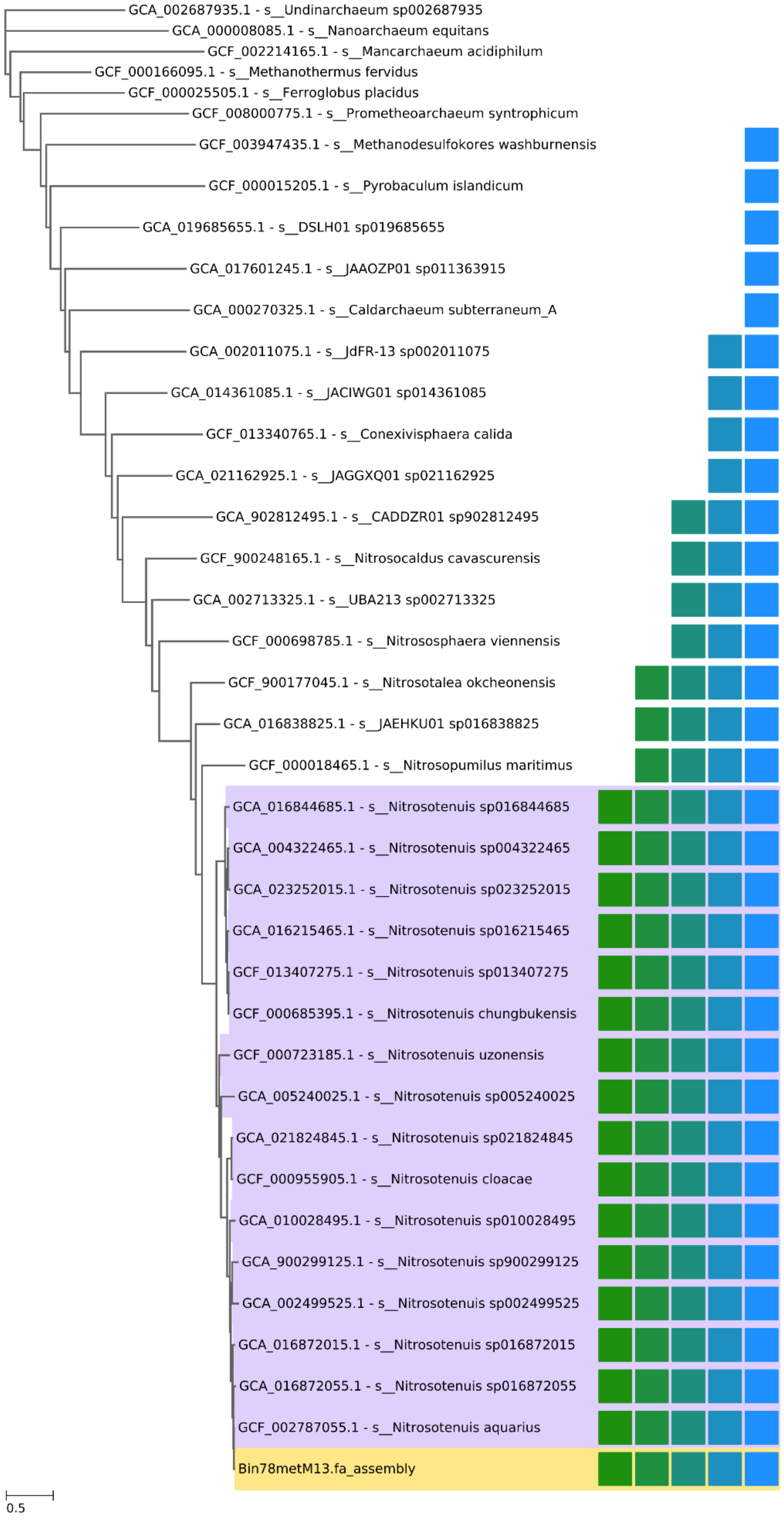

Figure S1. Genome-resolved phylogenomic classification of metagenome-assembled genomes (MAGs) using GTDB-Tk. Panels correspond to individual samples: (A) M5, (B) M6, (C) M8, (D) M9, (E) M11, and (F) M13. For each sample, the left phylogenetic tree summarizes archaeal MAG classifications, whereas the right tree summarizes bacterial MAG classifications. Colored labels indicate taxonomic assignments, and adjacent bar plots show the relative distribution of MAGs across lineages.

M5

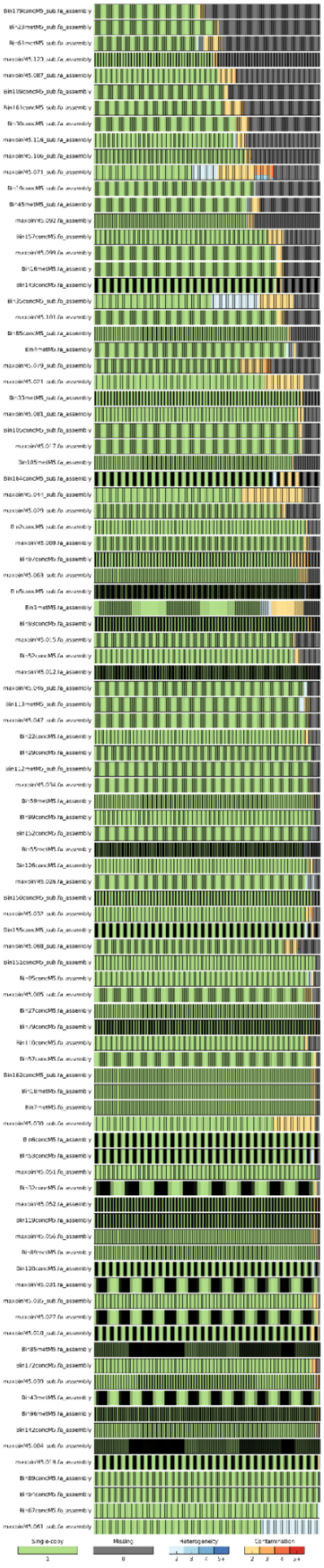
Single-copy
1
Missing
0
Heterogeneity
2
3
4
5+
Contamination
2
3
4
5+

M6

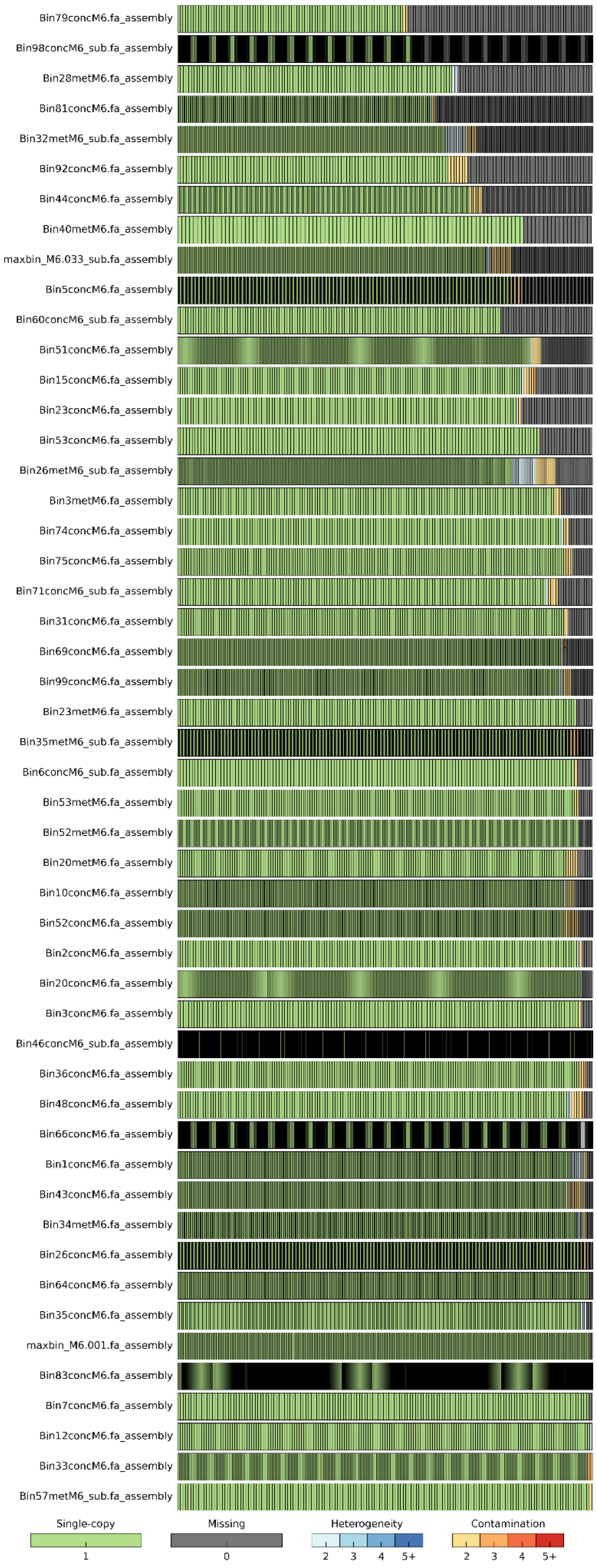
Bin79concM6.fa_assembly
Bin98concM6_sub.fa_assembly
Bin28metM6.fa_assembly
Bin81concM6.fa_assembly
Bin32metM6_sub.fa_assembly
Bin92concM6.fa_assembly
Bin44concM6.fa_assembly
Bin40metM6.fa_assembly
maxbin_M6.033_sub.fa_assembly
Bin5concM6.fa_assembly
Bin60concM6_sub.fa_assembly
Bin51concM6.fa_assembly
Bin15concM6.fa_assembly
Bin23concM6.fa_assembly
Bin53concM6.fa_assembly
Bin26metM6_sub.fa_assembly
Bin3metM6.fa_assembly
Bin74concM6.fa_assembly
Bin75concM6.fa_assembly
Bin71concM6_sub.fa_assembly
Bin31concM6.fa_assembly
Bin69concM6.fa_assembly
Bin99concM6.fa_assembly
Bin23metM6.fa_assembly
Bin35metM6_sub.fa_assembly
Bin6concM6_sub.fa_assembly
Bin53metM6.fa_assembly
Bin52metM6.fa_assembly
Bin20metM6.fa_assembly
Bin10concM6.fa_assembly
Bin52concM6.fa_assembly
Bin2concM6.fa_assembly
Bin20concM6.fa_assembly
Bin3concM6.fa_assembly
Bin46concM6_sub.fa_assembly
Bin36concM6.fa_assembly
Bin48concM6.fa_assembly
Bin66concM6.fa_assembly
Bin1concM6.fa_assembly
Bin43concM6.fa_assembly
Bin34metM6.fa_assembly
Bin26concM6.fa_assembly
Bin64concM6.fa_assembly
Bin35concM6.fa_assembly
maxbin_M6.001.fa_assembly
Bin83concM6.fa_assembly
Bin7concM6.fa_assembly
Bin12concM6.fa_assembly
Bin33concM6.fa_assembly
Bin57metM6_sub.fa_assembly
Single-copy
1
Missing
0
Heterogeneity
2 3 4 5+
Contamination
2 3 4 5+

M8

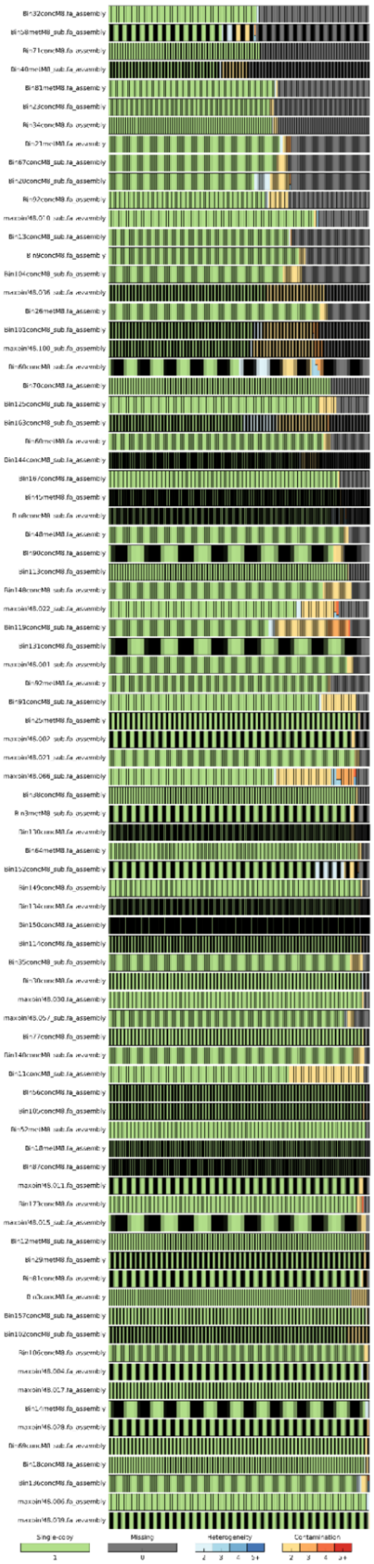

Single-copy
Missing
Heterogeneity
Contamination

M9

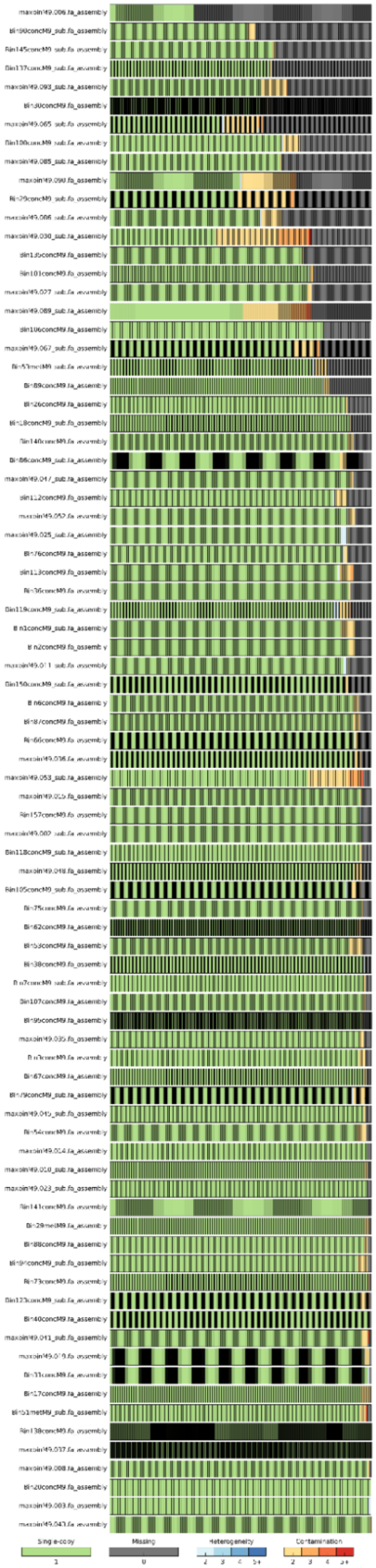
Single-copy
Missing
Heterogeneity
Contamination

M11

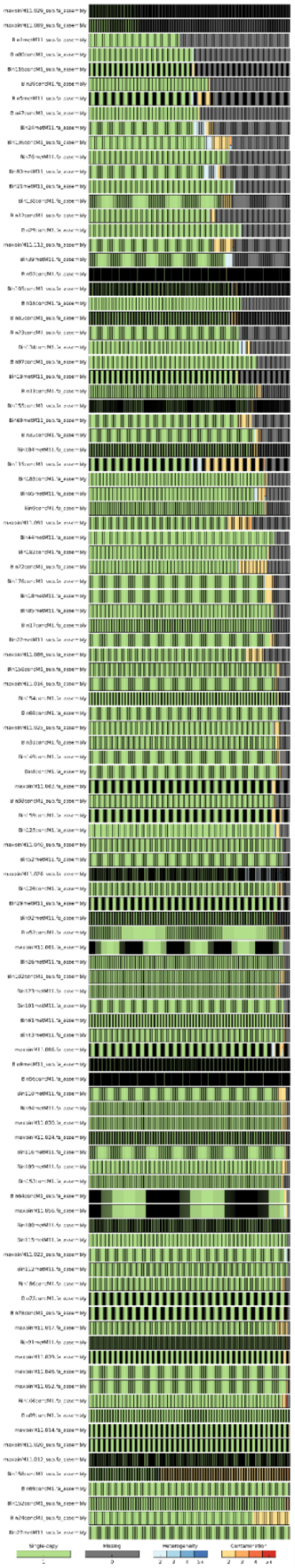
Single-copy
1
Missing
0
Heterogeneity
2
3
4
5+
Contamination
2
3
4
≥4

M13

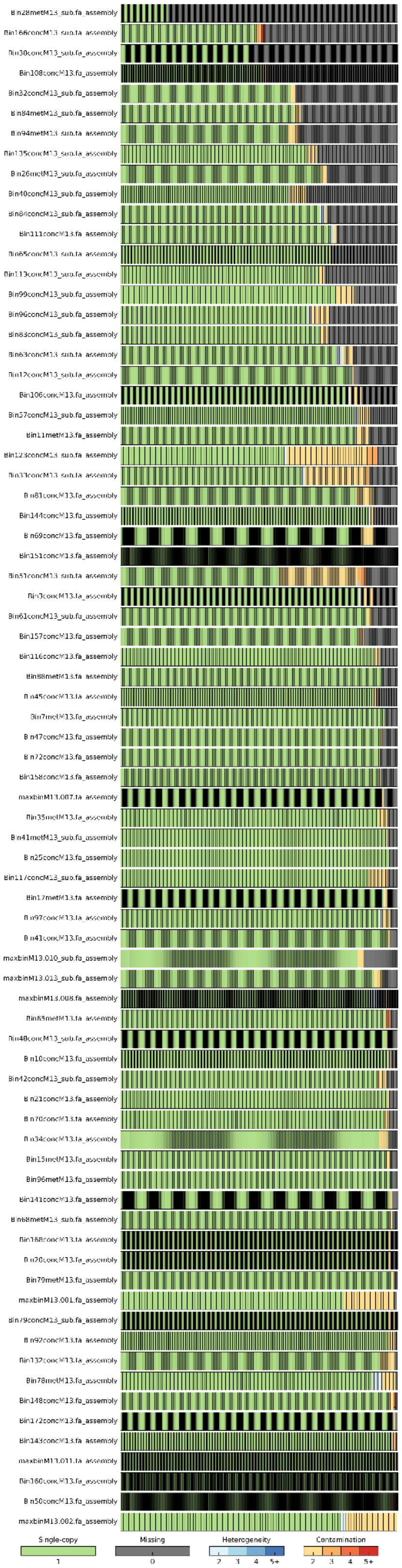
Bin28metM13_sub.fa_assembly
Bin166concM13_sub.fa_assembly
Bin30concM13_sub.fa_assembly
Bin108concM13.fa_assembly
Bin32concM13_sub.fa_assembly
Bin84metM13_sub.fa_assembly
Bin94metM13_sub.fa_assembly
Bin135concM13_sub.fa_assembly
Bin26metM13_sub.fa_assembly
Bin40concM13_sub.fa_assembly
Bin84concM13_sub.fa_assembly
Bin111concM13.fa_assembly
Bin65concM13_sub.fa_assembly
Bin113concM13_sub.fa_assembly
Bin99concM13_sub.fa_assembly
Bin96concM13_sub.fa_assembly
Bin83concM13_sub.fa_assembly
Bin63concM13_sub.fa_assembly
Bin12concM13_sub.fa_assembly
Bin106concM13.fa_assembly
Bin57concM13_sub.fa_assembly
Bin11metM13.fa_assembly
Bin123concM13_sub.fa_assembly
Bin33concM13_sub.fa_assembly
Bin81concM13.fa_assembly
Bin144concM13.fa_assembly
Bin69concM13.fa_assembly
Bin151concM13.fa_assembly
Bin51concM13_sub.fa_assembly
Bin3concM13.fa_assembly
Bin61concM13_sub.fa_assembly
Bin157concM13.fa_assembly
Bin116concM13.fa_assembly
Bin88metM13.fa_assembly
Bin45concM13.fa_assembly
Bin7metM13.fa_assembly
Bin47concM13.fa_assembly
Bin72concM13.fa_assembly
Bin158concM13.fa_assembly
maxbinM13.007.fa_assembly
Bin35metM13.fa_assembly
Bin41metM13_sub.fa_assembly
Bin25concM13.fa_assembly
Bin117concM13_sub.fa_assembly
Bin17metM13.fa_assembly
Bin97concM13.fa_assembly
Bin41concM13.fa_assembly
maxbinM13.010_sub.fa_assembly
maxbinM13.013_sub.fa_assembly
maxbinM13.008.fa_assembly
Bin85metM13.fa_assembly
Bin48concM13_sub.fa_assembly
Bin10concM13.fa_assembly
Bin42concM13_sub.fa_assembly
Bin21concM13.fa_assembly
Bin70concM13.fa_assembly
Bin34concM13.fa_assembly
Bin15metM13.fa_assembly
Bin96metM13.fa_assembly
Bin141concM13.fa_assembly
Bin68metM13_sub.fa_assembly
Bin168concM13.fa_assembly
Bin20concM13.fa_assembly
Bin79metM13.fa_assembly
maxbinM13.001.fa_assembly
Bin79concM13_sub.fa_assembly
Bin92concM13.fa_assembly
Bin132concM13.fa_assembly
Bin78metM13.fa_assembly
Bin148concM13.fa_assembly
Bin172concM13.fa_assembly
Bin143concM13.fa_assembly
maxbinM13.011.fa_assembly
Bin160concM13.fa_assembly
Bin50concM13.fa_assembly
maxbinM13.002.fa_assembly
Single-copy
1
Missing
0
Heterogeneity
2 3 4 5+
Contamination
2 3 4 5+

Figure S2. CheckM marker gene distribution across MAGs. Distribution of marker genes used by CheckM to estimate completeness and contamination across reconstructed metagenome-assembled genomes (MAGs). Each panel corresponds to a sample (M5–M13) and shows the presence of single-copy, duplicated, and missing marker genes used for genome quality assessment
